# Skull stripping tools in pediatric T2-weighted MRI scans: a retrospective evaluation of segmentation performance

**DOI:** 10.3389/fnins.2025.1715514

**Published:** 2025-12-18

**Authors:** Adrian Schulz, Eric Dragendorf, Katharina Wendt, André Schomakers, Eva Bültmann, Dominik Wolff

**Affiliations:** 1 Peter L. Reichertz Institute for Medical Informatics of TU Braunschweig and Hannover Medical School, Hannover Medical School, Hannover, Germany; 2 Institute of Diagnostic and Interventional Neuroradiology, Hannover Medical School, Hannover, Germany

**Keywords:** evaluation, skull stripping, magnetic resonance imaging, T2-weighted, pediatrics, dice score, retrospective studies

## Abstract

**Introduction:**

For brain maturity assessment of infants aged above 6 months, T2-weighted MRI scans are recommended. Prior to automated brain tissue analysis, skull stripping is typically applied. However, most skull stripping tools neither focus on T2-weighted scans nor on pediatric cohorts. Here, we present the evaluation results of seven common skull stripping tools in a comparably large pediatric cohort.

**Methods:**

This study is based on 199 T2-weighted scans of children under the age of 5 years retrospectively acquired from the clinical routine at Hannover Medical School. We established a manually labeled ground truth under quality control of a senior neuroradiologist specialized in pediatric neuroradiology and evaluated seven skull stripping tools (*BET*, *ROBEX*, *HD-BET*, *HD-BET-fast*, *SynthStrip*, *SynthStrip-noCSF* and *d-SynthStrip*). Segmentation performance (Dice score, 95th percentile Hausdorff distance, sensitivity, specificity) and computation time were assessed on non-preprocessed and preprocessed scans (zero padding, contrast enhancement, artifact removal and normalization) as well as in different brain regions. For the best performing model, we manually assessed the top and bottom quartile of segmentations with respect to the integrity of different anatomical brain structures.

**Results:**

Only *BET*, *HD-BET*, *HD-BET-fast* profited from data preprocessing. Considering this, all models had median Dice scores between 0.88 and 0.96, with *SynthStrip* performing best. All models segmented most accurately in the middle axial slices of the brain. Resampling lowered the performance of all models, except *ROBEX*. Mean computing times ranged from 2 s (*BET*) to 132 s (*HD-BET*) with *SynthStrip* requiring 7 s. per scan. *SynthStrip* was prone to not entirely including the *Sinus sagittalis superior*, the upper *Cerebrum*, the temporal pole, the *Cerebellum* and the *Chiasma opticum*/pituitary gland. In contrast, the petrous bone and the skull in the middle axial slices have often been partly included.

**Discussion:**

Due to its robustness and quick computation time, we recommend *SynthStrip* for skull stripping of pediatric T2-weighted MRI scans. We attribute the observed segmentation errors to the partial volume effect, which should be addressed in future research. Limitations of our study include the monocentric setting, the exclusion of pathological cases and the skewed age distribution in our cohort.

## 1 Introduction

By analyzing cerebral magnetic resonance images, an individual’s brain age can be derived. For children, the focus lays on the development of the brain (Morita et al., 2022). The maturation of the brain is a complex process involving structural and functional changes (Lenroot and Giedd, 2006; Stiles and Jernigan, 2010) that correlate with an infant’s development. For tracking anatomical brain changes, such as myelination status as well as changes in volume and cortical thickness, magnetic resonance imaging (MRI) is the imaging modality of choice (Allen et al., 2005; Stiles and Jernigan, 2010). Dragendorf et al. (2024) suggested the automation of pediatric brain age prediction based on MRI scans in the clinical routine through the application of artificial intelligence (AI). For this, skull stripping is a crucial preprocessing step (Bernal et al., 2019; Fatima et al., 2020). Skull stripping, also referred to as brain extraction, removes non-brain tissue from scans. This way, potential age-related covariates, like the skull thickness in children, are removed, which allows age predictions solely based on relevant tissue.

Over several decades, a variety of skull stripping methods have been introduced. There has been a shift from the initial conventional image-processing-based methods toward machine learning (ML) and deep learning (DL) (Fatima et al., 2020). The conventional methods can be further subcategorized into five groups (Kalavathi and Prasath, 2016): mathematical morphology-based, intensity-based, deformable surface-based, atlas/template-based and hybrid models. According to Bernal et al. (2019), two frequently used conventional skull stripping tools are *BET* and *ROBEX*.

*BET* (Brain Extraction Tool) belongs to the group of deformable surface-based models, which performs skull stripping in a two-step approach (Smith, 2002). It identifies the center of the brain and, from there, grows a triangle mesh until it reaches the brain’s contours. For finding the center, *BET* applies a histogram-derived binarization threshold leading to a rough separation of the head from the background. Within the binarized volume, the center of gravity (COG) is calculated, which approximately corresponds to the brain’s center. For initializing a spherical triangle mesh around the COG, a reasonably small radius must be defined that allows the mesh to grow and, thus, adapt to the brain’s surface from the inside of the organ. To determine this radius, a sphere centered at the COG is fitted to the head voxels of the binarized scan. Since it risks exceeding the brain’s boundaries, its radius is halved. The result is a small sphere in the middle of the brain, that serves as the initialization of a mesh within the non-binarized scan. Growing the mesh is an iterative process of minimally altering the triangles’ tips (vertices) until they reach voxels with intensities below a locally calculated threshold. By this, *BET* relies on the change in intensity at the boundary from brain to cerebrospinal fluid (CSF).

*ROBEX* (Robust, Learning-Based Brain Extraction System) is considered a conventional hybrid tool (Kalavathi and Prasath, 2016), although it combines two ML models: a Random Forrest Classifier (RFC) as a discriminative model and a Point Distribution Model (PDM) as a generative model (Iglesias et al., 2011). For each voxel in an intensity-standardized scan, the RFC determines whether it is part of the brain outline, thus creating a single brain shape within a scan. Ten voxel features, which are linked to the voxels’ location (x-, y-, and z-coordinates) and intensity context (application of edge detection filters), serve as input to the RFC. The resulting brain shape is highly individual but might overshoot and not always be realistic. In contrast to the RFC, the PDM can generate a multitude of brain shapes by deforming a mean brain shape established from training data. The degree of deformation is restricted, which on the one hand prevents the PDM from creating unrealistic shapes, but on the other hand also prevents the shapes from perfectly matching an individual brain. *ROBEX* finds a compromise between the two ML methods by fitting a PDM shape to the RFC shape.

In contrast to conventional methods, such as *BET* and *ROBEX*, there are more modern approaches based on DL, such as *HD-BET* (Isensee et al., 2019). Its core component is a special type of convolutional neural net, the U-Net (Ronneberger et al., 2015). U-Net consists of a contracting path and an expansive part that are connected through skip-connections resulting in a u-shaped network scheme. The contracting path abstracts the image to its semantic essence by applying convolutions and thus reducing the feature map size. The gain in semantic information is paid for with a loss of localization information. In the expansive path, feature maps are enlarged again while adding previously lost localization information via the skip-connections to generate the image mask. During the training of *HD-BET* with a total of 1,500 scans, a five-fold cross-validation was performed, which resulted in five different U-Net models forming an ensemble for the brain mask prediction. *HD-BET* applies test time augmentation (TTA). This technique uses modified copies of the input image and their associated predicted masks to finetune and generate the final brain mask. As an alternative to the U-Net ensemble with TTA, *HD-BET* can be limited to using a single U-Net without TTA (referred to as *HD-BET-fast* in this paper).

*SynthStrip* is another U-Net-based model for skull stripping (Hoopes et al., 2022). Compared to *HD-BET*, it has a fundamentally different approach concerning its training data. With 80 T1-weighted MRI scans, only a very limited set of training data was used. But the data was vastly augmented during model training. The dynamic augmentation included translation, rotation, scaling and non-linear deformation to different degrees, which were randomly performed in each epoch. Further, 46 distinct anatomical regions within the scan were assigned randomly chosen voxel intensities and bias fields were introduced to varying extents. Additionally, the overall brightness of the scans was altered, and a Gaussian blur was introduced. Moreover, half of the time, the scans were randomly cropped and down sampled. This data augmentation strategy even led to unrealistic training images with the aim of creating a robust model for various input modalities. A specialized pediatric version, *developmental SynthStrip* (*d-SnythStrip*), was trained on a mixture of 57 T1-weighted (T1W) and T2-weighted (T2W) scans of children under the age of 4.5 years (Kelley et al., 2024). *SynthStrip* includes CSF in its brain masks by default. This behavior can be adapted for receiving segmentations without CSF (referred to as *SynthStrip-noCSF*). The immutable default of *d-SynythStrip* does not include any CSF in the brain masks.

Although required in the context of brain maturity assessment in children, few of the available skull stripping tools target pediatric populations (Das et al., 2023; Kelley et al., 2024). More specifically, for brain age predictions in children over the age of 6 months, T2-weighted MRI scans are recommended (Barkovich and Barkovich, 2018). However, the brain stripping tools mentioned above have not been evaluated on T2W pediatric scans. The only exception is *d-SynthStrip*, whose testing dataset included 39 scans of that kind. Rehman et al. (2020) pointed out that the performance of brain extraction tools may deteriorate when applied to different datasets and that there was a need for tools suited for modalities such as T2W scans.

Here, we investigate in a pediatric cohort the performance of seven open-source skull stripping models (*BET*, *ROBEX*, *HD-BET*, *HD-BET-fast*, *SynthStrip*, *SynthStrip-noCSF*, *d-SynthStrip*). For this, we use 199 clinical routine T2W MRI scans of children under the age of 5 years with normal brain development retrospectively retrieved at Hannover Medical School. By analyzing both the overall and regional segmentation performance, we provide researchers and clinical users in the field with a reliable recommendation. To our knowledge, there is yet no such evaluation, while other studies have focused on different aspects, such as neonatal populations (Vaz et al., 2024) or T1W scans (Das et al., 2023).

## 2 Materials and methods

### 2.1 Cohort

**Figure 1:**
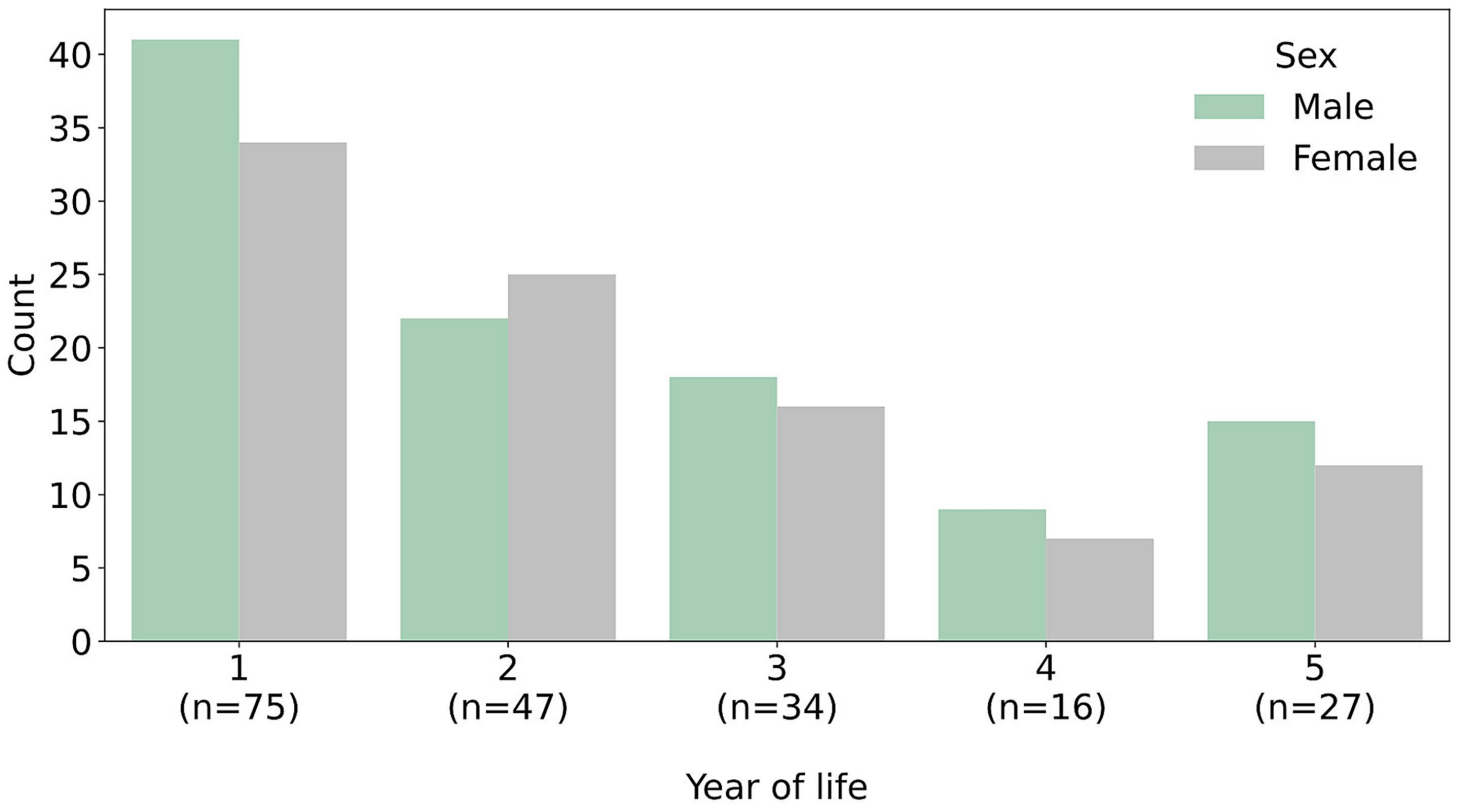
Distribution of age and sex within the cohort (*n* = 199).

We acquired all available T2W MRI scans matching the inclusion criteria from the clinical routine at the Institute of Diagnostic and Interventional Neuroradiology of Hannover Medical School (MHH) between 2011 and 2023. Inclusion criteria were normal brain development, absence of any pathologies, age under 5 years and parental informed consent to the secondary data use for research purposes. Scans were checked by a senior neuroradiologist specialized in pediatric neuroradiology (EB) and excluded in case of relevant motion artifacts. The cohort included 199 children (94 female, 105 male) who had been scanned for the assessment of their brain development in general anesthesia or sedation. The age range in the cohort spanned from four to 1791 days (4.9 years) with a median age of 1.4 years, a mean (*M*) of 1.8 years and a standard deviation (*SD*) of 1.4 years (Figure 1). The research was approved by the ethics committee at MHH (Nr. 11658_BO_K_2024).

### 2.2 MRI protocol

All scans were T2W scans with from either a *Siemens Magnetom Verio* 3 Tesla or a *Siemens Avanto* 1.5 Tesla MRI scanner. Due to the infants differing head sizes, the number of axial slices ranged from 24 to 44. Since all data originate from the clinical routine there is no consistent protocol. 162 scans had a voxel size of 0.43 mm × 0.43 mm × 4.00 mm and 4.00 mm spacing between slices. The remaining 37 scans had varying voxel dimensions and slice spacings (Supplementary Table A). Echo times ranged from 127 ms to 131 ms, repetition times from 2,860 ms to 9,010 ms, and flip angles from 120° to 150°.

### 2.3 Data preparation

**Figure 2:**
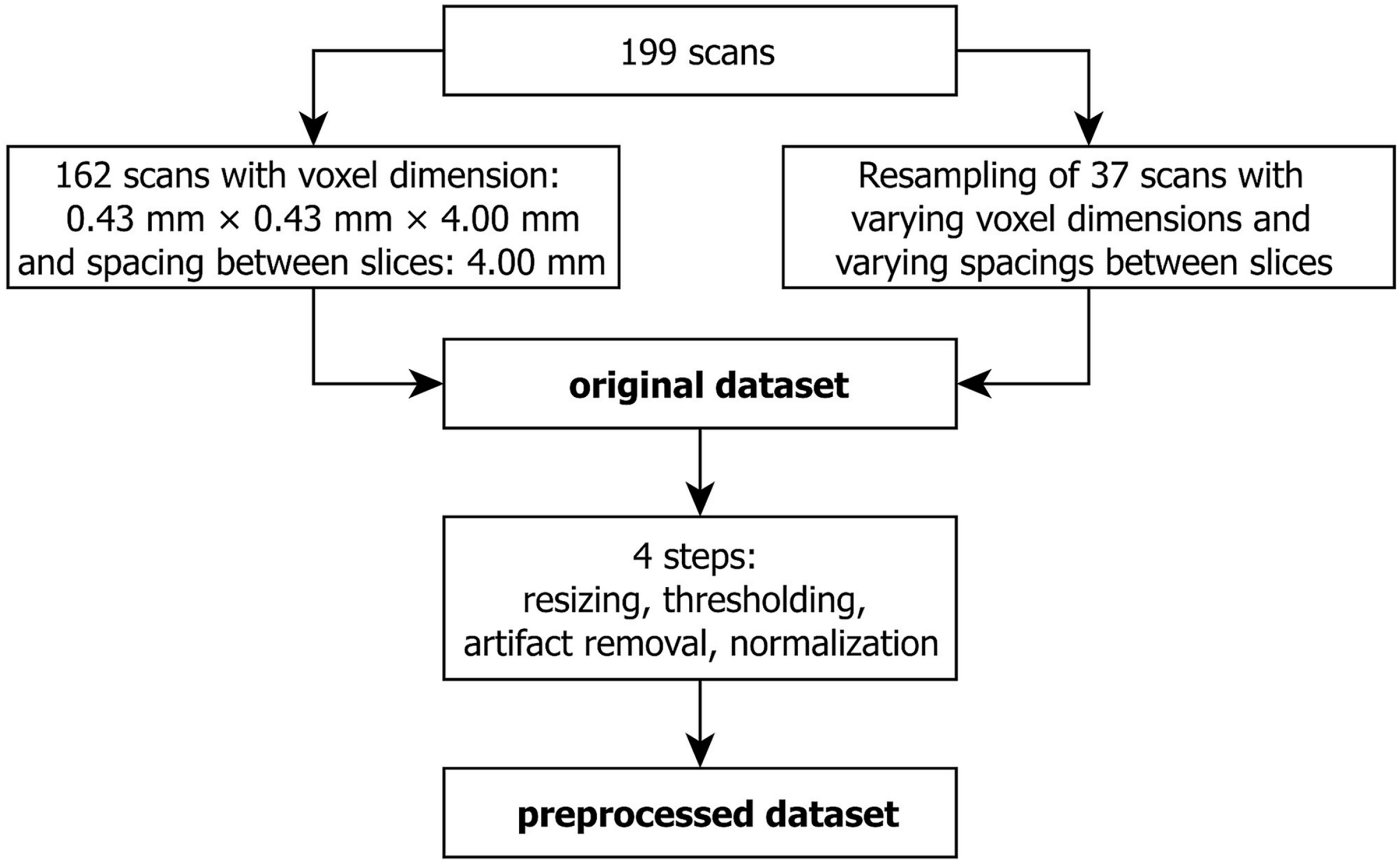
Curation of the original and the preprocessed dataset from the same 199 scans.

We extracted the MRI scans from the PACS at MHH as DICOM files and converted them to NIFTI format with dcm2niix (version v1.0.20211006). Using Lanczos3 as a resampling filter from MeVisLab (version 3.7.0.14), we resampled the 37 scans with varying dimensions according to the voxel size and slice spacing of the other 162 scans (Figure 2). Based on these unified scans, referred to as “original” in this paper, we performed manual segmentation.

**Figure 3:**
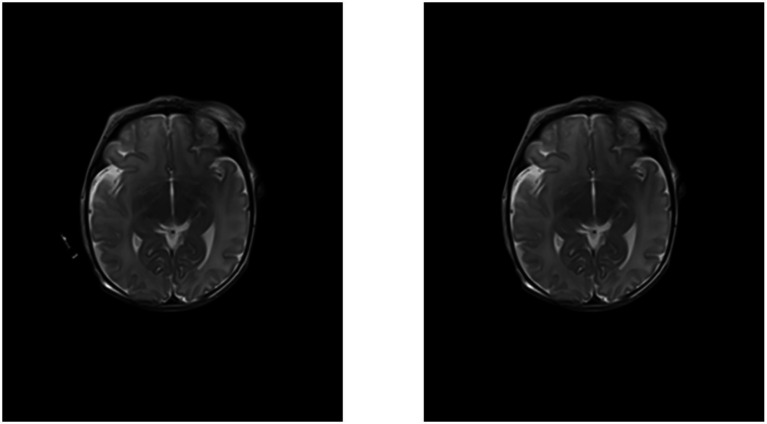
Axial slice of a T2-weighted MRI scan before (left) and after (right) artifact removal.

Data preprocessing was implemented in Python (version 3.12) and included four steps: resizing, contrast enhancement, artifact removal and normalization. By zero padding, we adjusted all scans to the size of the largest scan (484 voxels × 596 voxels × 40 voxels). For contrast enhancement, we applied an image specific threshold based on the triangle method. We handled artifacts by detecting objects through region property detection and only keeping the largest object in the image, which is the patient’s head including the skull (Figure 3). Finally, we min-max normalized the scans.

### 2.4 Ground truth

A medical student (ED) performed manual labeling of the scans using 3D Slicer (version 5.4.0) (Fedorov et al., 2012) and a neuroradiologist specialized in pediatric neuroradiology (EB) quality checked all manual segmentations, which were, if necessary, refined accordingly. The segmentation process was carried out in the axial plane according to the following rules. The segmentation of the outer boundary covers the entire cranial content. Therefore, voxels with partial volume sections (e.g., bone and parenchyma) are included. The cranial part of the S*inus sagittalis superior* is only included if brain parenchyma is present within the slice. The frontobasal area, including the CSF around the olfactory nerve, is segmented. Further, all components of the *Sinus durae matris* and spaces containing CSF are included. The caudal segmentation includes the entire *Cerebellum*. The *Medulla oblongata* is included down to the level of the caudal edge of the cerebellar tonsils.

### 2.5 Segmentation models

In this paper, we assess seven open source skull stripping models, namely *BET*^1^ (version 6.0.7.14 as part of the FSL library), *ROBEX*^2^ (version 1.2), *HD-BET* and *HD-BET-fast*^3^ (both version 1.0), as well as *SynthStrip*, *SynthStrip-noCSF* and *d-SynthStrip*^4^ (as part of Freesurfer version 7.4.1, the *pth*-file containing weights and biases for *d-SynthStrip* was manually downloaded on December 17, 2024), on both the original and the preprocessed data. We ran all models on a NVIDIA A100 GPU with 40 GB VRAM on a Rocky Linux System (version 9.4). For all models, we monitored the computing time per scan.

### 2.6 Evaluation

For comparing the automated segmentations (S) with the manually established ground truth (GT), we calculated the Dice score, 95th percentile Hausdorff distance (HD_95_), sensitivity and specificity as evaluation metrics. Using the Dice score and HD_95_, we evaluated for each model whether data preprocessing should be applied. Based on that, we compared the models’ performance (Dice score and HD_95_) in whole scans and applied statistical testing. We used the Shapiro–Wilk test to check for normality in the metrics’ distributions. Then, we applied the Friedman test as a non-parametric test for related samples. A two-sided Wilcoxon signed-rank test served as a post-hoc test. The significance level was set to *α* = 0.05, and the *p*-values were adjusted based on the Bonferroni correction. For comparing the best with all competing models, we visualized the respective Dice score differences in Bland–Altman plots. Analog to Vaz et al. (2024), we analyzed the evaluation metrics in different brain regions by splitting the scans. With their 22 neonates, Vaz et al. (2024) defined fixed slice number ranges to distinguish three regions along the longitudinal scan axis. Since our cohort contains children of different ages and, therefore, scans with varying slice numbers, we based our splits on the dimensions of the corresponding ground truth masks. In the longitudinal direction, we performed three splits of equal size (superior, central and inferior axial slices). Additionally, we divided the brain along the transversal axis into its left and right sagittal slices. Along the sagittal axis, it was subdivided into the anterior and posterior coronal slices. In addition, a medical student (ED) and a physician (AS) manually assessed the integrity of different anatomical structures in the top and the lowest quartile of segmentations of the best performing model with 3D Slicer (Fedorov et al., 2012). Finally, we evaluated resampling, sex and age as potential covariates for the models’ overall performance. For each model, Dice score and HD_95_ distributions of the resampled and non-resampled cases were tested for normality with a Shapiro–Wilk test. Subsequently, we used the two-sided Brunner Munzel test as a non-parametric test, which tolerates unequal sample sizes to test Dice scores as well as HD_95_ in the 37 resampled scans against the 167 non-resampled ones for each model. The significance level was set to *α* = 0.05, and the *p*-values were adjusted based on the Bonferroni correction. The correlation of age and segmentation performance was quantified using the Pearson correlation coefficient (PCC) with |PCC| > 0.1 indicating a weak, |PCC| > 0.4 indicating a moderate and |PCC| > 0.7 indicating a strong correlation (Schober et al., 2018). For comparison, the Dice scores of each model were visualized as violin plots. The violins’ range is limited to the actual data range and the violins’ maximum width is constant. All calculations and visualizations were performed with Python (version 3.11). We use the reporting guideline STARE-HI (STAtement on the Reporting of Evaluation studies in Health Informatics) by Talmon et al. (2009) and provide the checklist in the supplements.

## 3 Results

**Figure 4:**
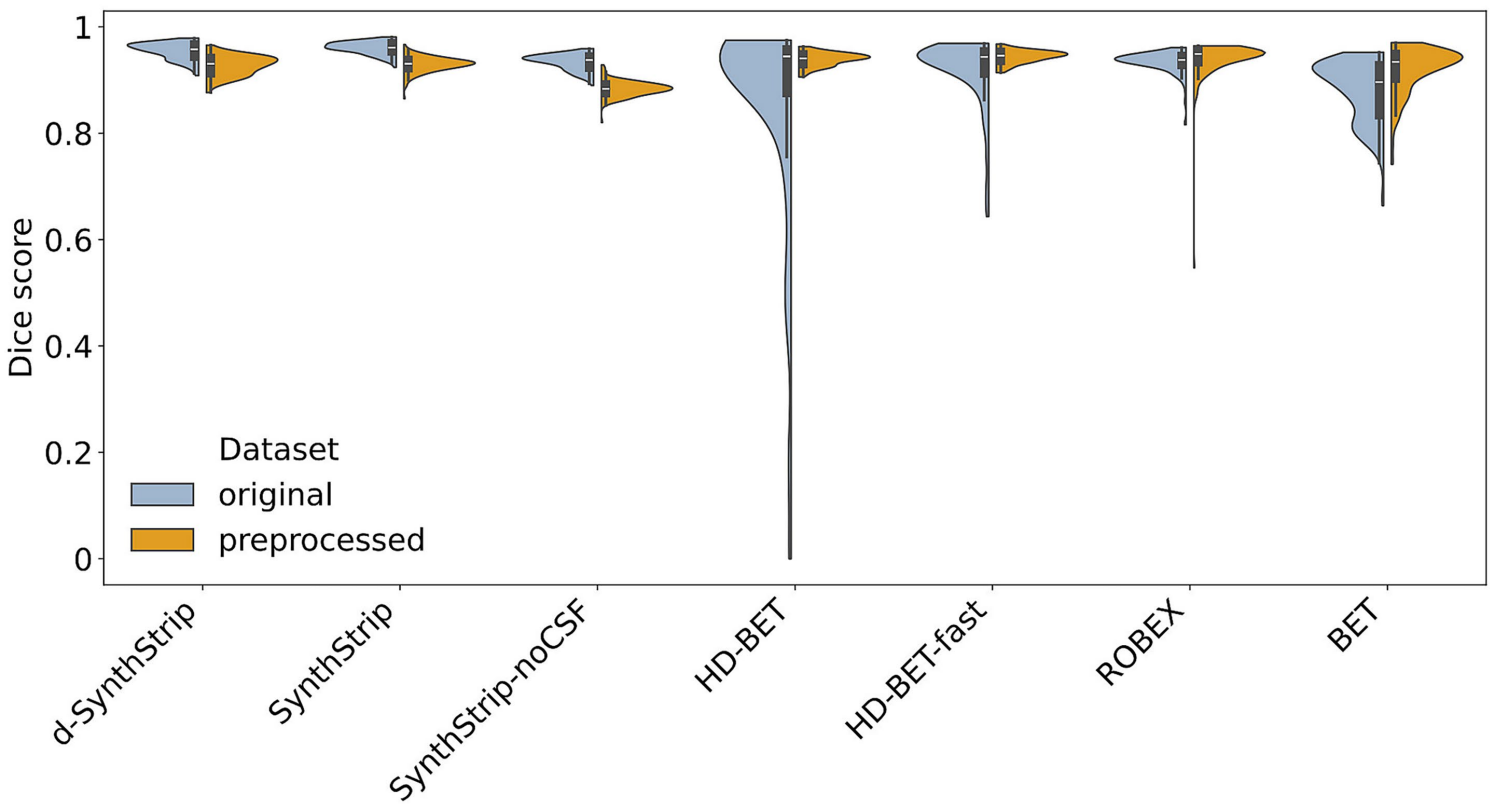
Performances of skull stripping models (Dice score) based on the original and the preprocessed scans of all 199 cases.

A total of seven skull stripping models were evaluated on the original as well as the preprocessed dataset. We compared their segmentation results to the manually labeled ground truth by calculating different evaluation metrics (Figure 4 and Supplementary Table B). Median Dice scores were between 0.884 and 0.961, while the value ranges showed larger differences, especially due to data preprocessing.

### 3.1 Data preprocessing influences the models’ segmentation results

Data preprocessing had varying effects on the models’ performances. *BET* was positively affected (*Δ* median Dice score = 0.038, Δ median HD_95_ score = −3.7 mm) including a narrower value range in both metrics. Although both *HD-BET* and *HD-BET-fast* had slightly higher median Dice scores and a smaller median HD_95_ on the original dataset, their value ranges drastically improved through preprocessing. The positive influence of preprocessing on *ROBEX* was limited to the median Dice score, whereas the HD_95_ and the respective value ranges deteriorated. Preprocessing negatively affected all of *SynthStrip*-related models, lowering their median Dice score by up to 0.054 and increasing the median HD_95_ by 2.9 mm – 8.0 mm, while broadening their respective value ranges. Thus, only three models, namely *BET*, *HD-BET-fast* and *HD-BET*, were improved by data preprocessing.

### 3.2 Skull stripping models differ in their segmentation performance

To compare the skull-stripping models, we used each model’s best-performing data preprocessing configuration (original vs. preprocessed dataset). *SynthStrip* achieved the highest median Dice score (0.961, [0.924–0.981]) and the smallest median HD_95_ (4.0 mm, [2.3 mm – 8.0 mm]) followed by *d-SynthStrip*. The lowest median Dice score (0.934, [0.742–0.970]) and the largest median HD_95_ (12.3 mm, [4.0 mm – 44.4. mm]) were observed using *BET*. *ROBEX* also had a low Dice score performance. In contrast, concerning their sensitivity, *ROBEX* and *BET* had the highest median values (0.993 and 0.942), whereas *SynthStrip-noCSF* and *HD-BET* were the least sensitive models (0.885 and 0.892). The median specificity of all models was remarkably high with *ROBEX* and *BET* being the only models below 0.99.

**Figure 5:**
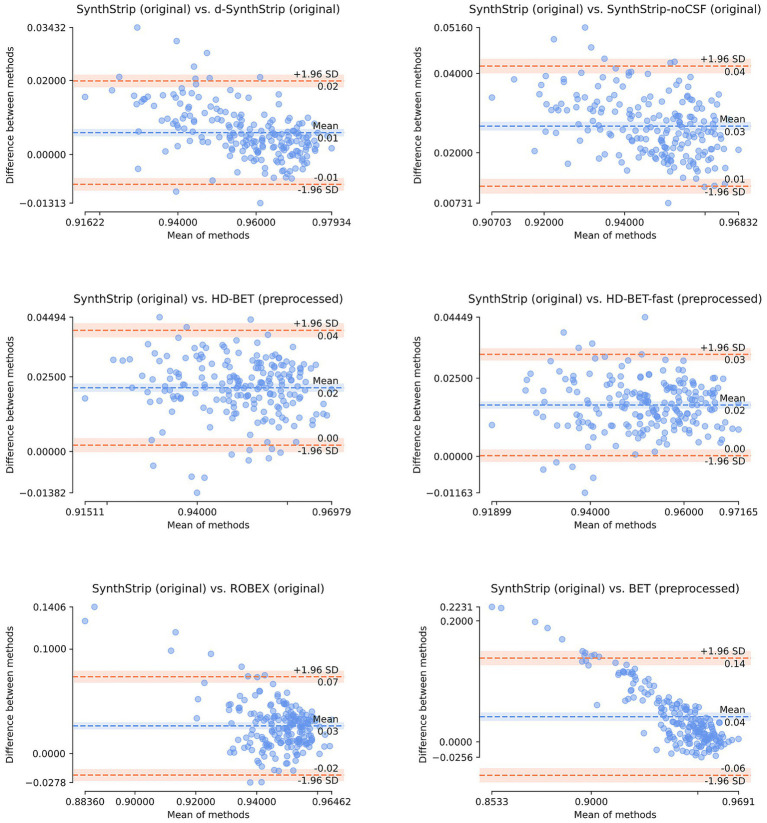
Bland–Altman plots comparing the segmentation performance (Dice score) of *SynthStrip* on all 199 cases to the segmentation performances of the six other skull stripping models.

All model comparisons apart from three exceptions (*SynthStrip-noCSF* vs. *BET*, *ROBEX* vs. *SynthStrip-noCSF*, *ROBEX* vs. *HD-BET*) showed statistically significant Dice score and HD_95_ differences (Supplementary Table C). Thus, the performance advantage of *SynthStrip* over all other tools is most likely not based on chance. Bland–Altman plots (Figure 5) reveal, that the elevation of *SynthStrip’s* mean Dice score by 0.01 to 0.04 in comparison to the other skull stripping models is also reflected on an individual level. *BET* and *d-SynthStrip* achieved higher Dice scores in 17% of the cases and the remaining models outperformed *SynthStrip* in only under 10% of the scans. The margin by which models outperformed *SynthStrip* in these cases was small compared to the margin in the majority of cases, where they underperformed. When comparing *SynthStrip* to *BET* as well as *ROBEX*, this margin clearly increased with the decrease in combined mean performance. This tendency is also visible in the comparison to d-*SynthStrip* and *SynthStrip-noCSF*.

With a mean computation time per scan of 7 s (*SD* = 0.90), *SynthStrip* was among the faster models with only *BET* being considerably quicker (*M* = 2.4 s, *SD* = 0.62), whereas *ROBEX* (*M* = 40.8 s, *SD* = 2.38) and *HD-BET* (*M* = 132 s, *SD* = 0.83) had a substantially higher computing time (Table 1).

**Table 1:** Descriptive statistics of the models’ computing time in seconds over all 199 cases.

Model (dataset)	*M*	SD	Median	Min	Max
*BET* (preprocessed)	2.39	0.62	2	1	4
*SynthStrip-noCSF* (original)	7.01	0.88	7	6	10
*d-SynthStrip* (original)	7.02	0.89	7	6	10
*SynthStrip* (original)	7.09	0.90	7	6	11
*HD-BET-fast* (preprocessed)	8.41	0.49	8	8	9
*ROBEX* (original)	40.8	2.38	41	34	47
*HD-BET* (preprocessed)	131.81	0.83	132	130	139

### 3.3 Regional segmentation performances

**Figure 6:**
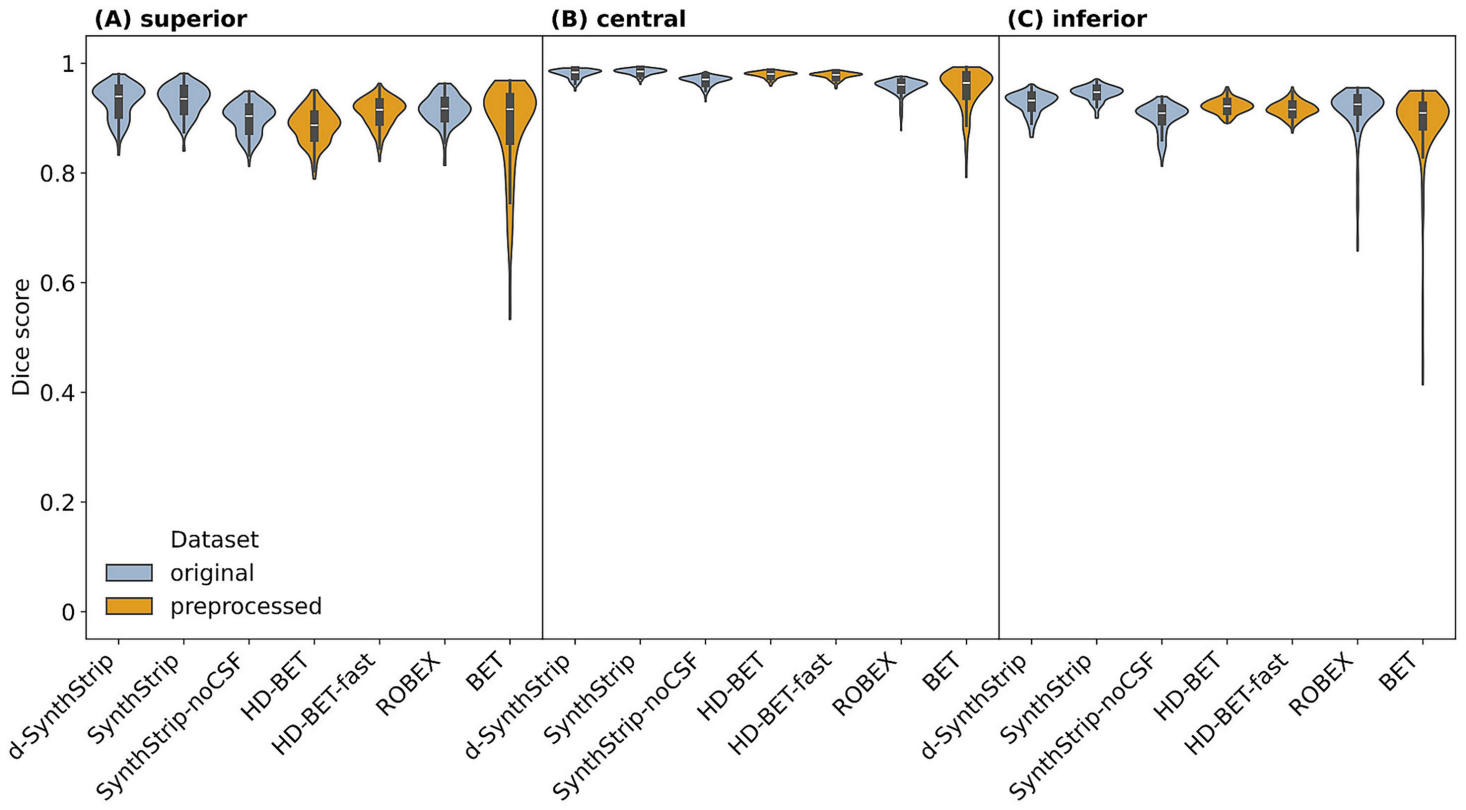
Regional skull stripping performances (Dice score) along the longitudinal scan axis in the superior (**A**), central (**B**) and inferior region (**C**).

Along the longitudinal scan axis, all models performed better in the central region compared to the inferior one (*Δ* median Dice scores = [0.036–0.063], Δ median HD_95_ = [−3.2 mm – −0.4 mm]). With the single exception of *BET*, this was also observed when comparing the central to the superior region (Figure 6 and Supplementary Table D). In the central region, all models’ median Dice scores lay above 0.961 and the median HD_95_ below 8.0 mm with *SynthStrip* achieving the best values (median Dice score = 0.985, median HD_95_ = 1.4 mm). The comparison of the left and right hemisphere (Supplementary Figure E and Supplementary Table E) did not reveal any considerable differences (Δ median Dice scores ≤ 0.01, Δ median HD_95_ ≤ 1.0 mm). The only model clearly showing better values for both the Dice score and the HD_95_ in the posterior region compared to the anterior one was *SynthStrip-noCSF* (Δ median Dice scores = 0.036, Δ median HD_95_ = 3.3 mm) (Supplementary Figure F and Supplementary Table F).

**Figure 7:**
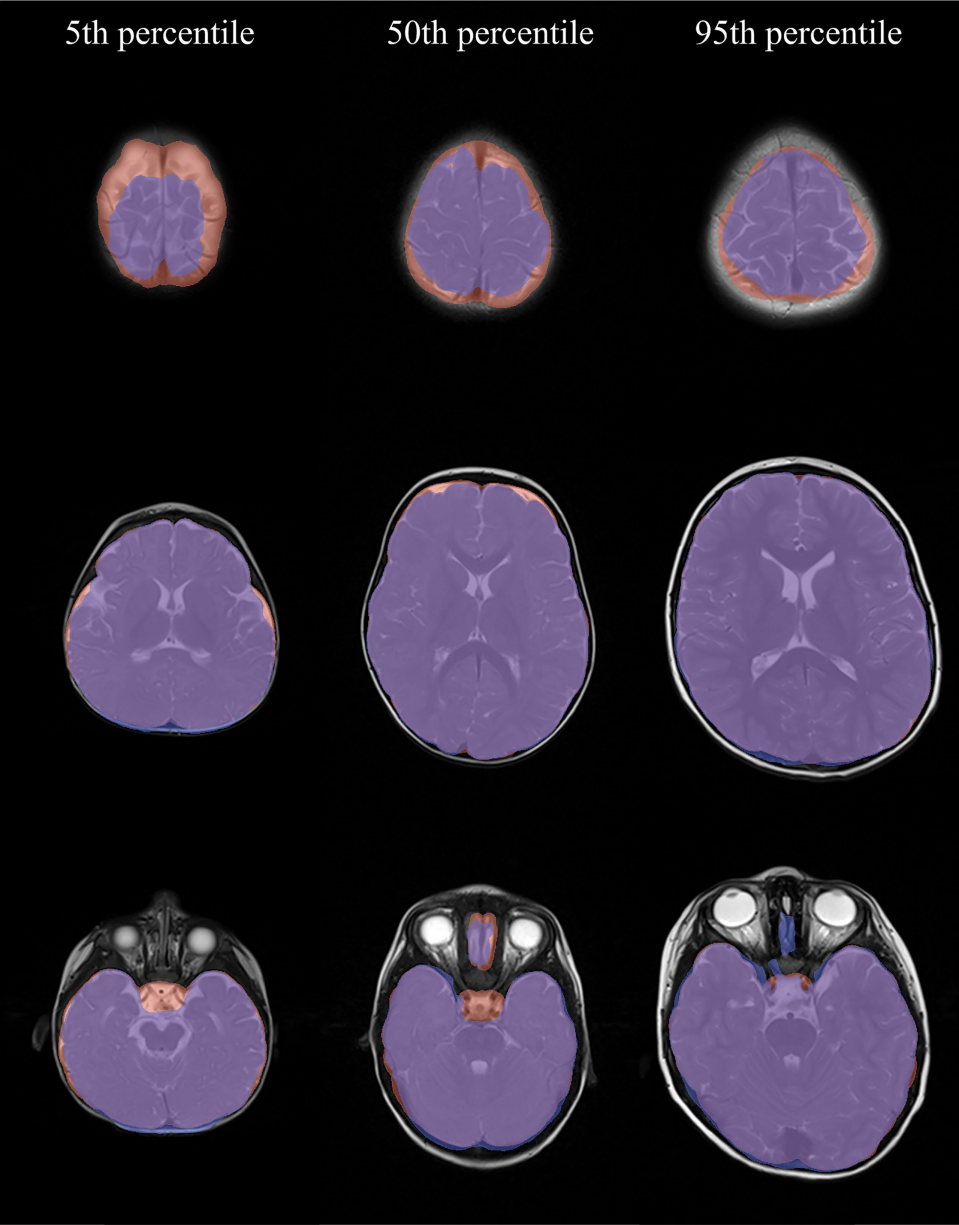
Exemplary *SynthStrip* segmentations (blue) and the corresponding ground truth (red). The percentiles refer to the Dice score distribution of all 199 cases.

Regarding the best performing model, *SynthStrip*, in about one third of the highest and lowest quartiles of segmentations, the *Cerebellum* was not entirely detected (Table 2). In about two thirds of these cases, the *Sinus sagittalis superior*, the *Chisma opticum*/pituitary gland and the pole of the temporal lobe were partially missing. With only four exceptions, the upper axial slices of the cerebrum were not fully segmented (Figure 7). In the central slices in over 90% of these cases, anterior and lateral CSF was missing, whereas parts of the posterior and lateral skull were included. Parts of the petrous bone were also included in 81%.

**Table 2:** Manual assessment of multiple anatomical brain structures in the highest and lowest quartiles of *SynthStrip* segmentations with respect to the Dice score.

Anatomical region	Anatomical structure	Criterion	Highest quartile (*n* = 50)	Lowest quartile (*n* = 50)	Highest and lowest quartiles (*n* = 100)
Superior	*Sinus sagittalis superior*	Complete Inclusion (yes/no)	33/17	3/47	36/64
	Upper *Cerebrum*	Complete Inclusion (yes/no)	4/46	0/50	4/96
Center	Skull	Partial Inclusion (yes/no)	46/4	45/5	91/9
	CSF	Complete Inclusion (yes/no)	3/47	0/50	97/3
Inferior/Anterior	*Chiasma opticum*/pituitary gland	Complete Inclusion (yes/no)	28/22	1/49	29/71
	Temporal pole	Complete Inclusion (yes/no)	35/15	2/48	37/63
	Petrous bone	Partial Inclusion (yes/no)	46/4	35/15	81/19
Inferior/Posterior	*Cerebellum*	Complete Inclusion (yes/no)	44/6	21/29	65/35

### 3.4 Potential covariates

**Figure 8:**
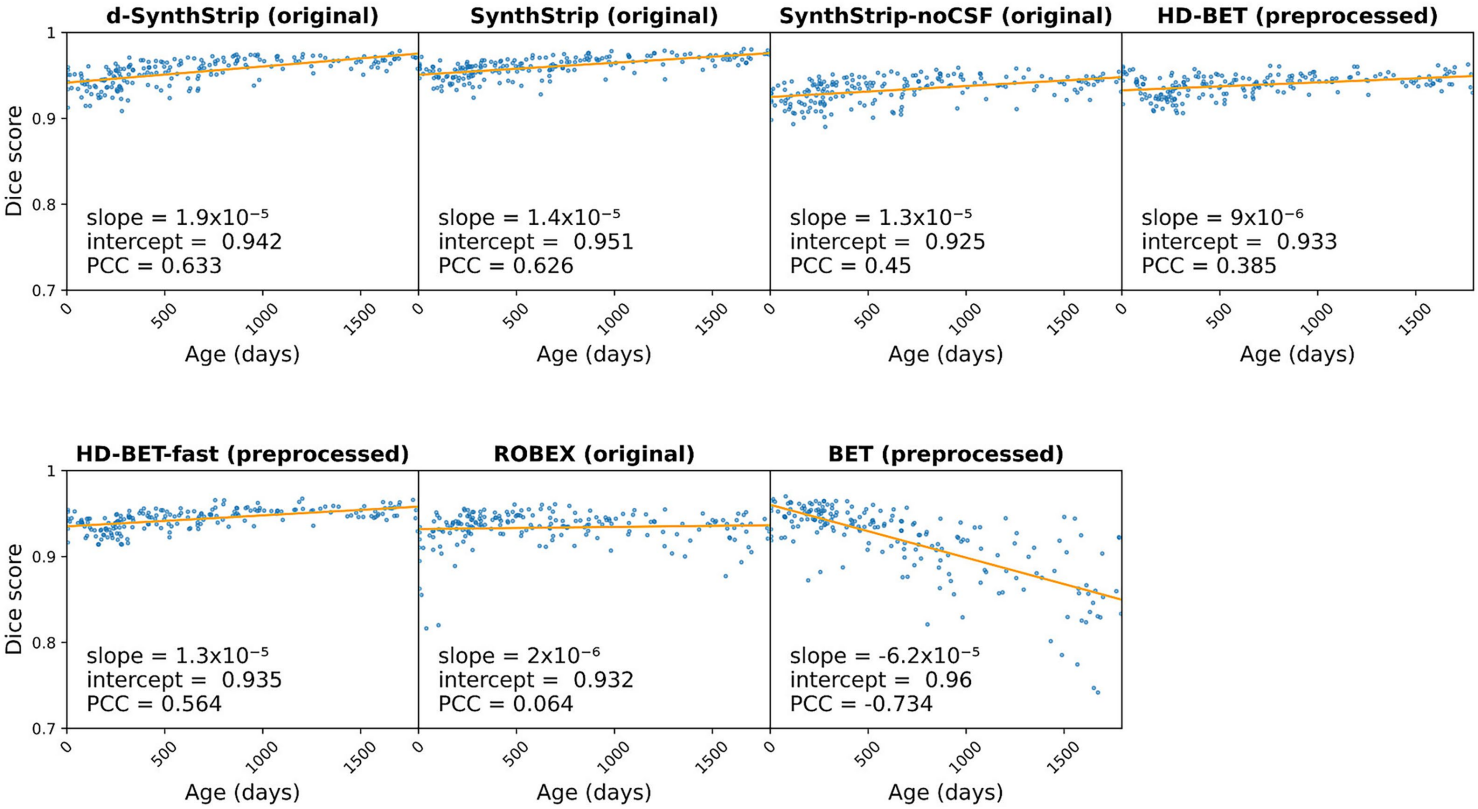
Linear regression analysis of segmentation performance (Dice score) in relation to infants’ age for all 199 cases (PCC: Pearson correlation coefficient).

All *SynthStrip*-related models dropped statistically significant in their segmentation performance regarding both Dice score and HD_95_ when comparing the 162 non-resampled scans to the 37 resampled ones (Table 3 and Supplementary Figure G and Supplementary Table G). For *HD-BET-fast* only the Dice score difference was statistically significant. Sex did not alter the segmentation performance of any model (Supplementary Figure H and Supplementary Table H). The segmentation performance of *BET* showed a strong correlation with the covariate age with a PCC of −0.734 (Figure 8). Its median Dice score gradually decreased from 0.950 under the age of one until it was 0.857 during the fifth year of life (Supplementary Table J). Contrarily, all *SynthStrip*-related models as well as *HD-BET-fast* showed a performance increase of 0.015 to 0.027 over the same age span with a moderate correlation (PCCs between 0.564 and 0.633).

**Table 3:** Segmentation performance metrics on 37 resampled MRI scans and 162 non-resampled scans.

Model (dataset)	Resampling	Dice score Median (min-max) *M* (SD)	Sensitivity Median (min-max) *M* (SD)	Specificity Median (min-max) *M* (SD)	HD_95_ Median (min-max) *M* (SD)
*BET* (preprocessed)	True	0.93 (0.774–0.967) 0.916 (0.044)	0.948 (0.656–0.985) 0.907 (0.081)	0.985 (0.959–0.997) 0.984 (0.007)	11.2 (4.0–44.4) 14.3 (9.3)
*BET* (preprocessed)	False	0.934 (0.742–0.97) 0.919 (0.043)	0.94 (0.635–0.99) 0.912 (0.078)	0.981 (0.933–0.996) 0.98 (0.009)	12.7 (4.0–32.9) 13.9 (7.5)
*HD-BET* (preprocessed)	True	0.931 (0.906–0.957) 0.935 (0.013)	0.875 (0.829–0.952) 0.884 (0.028)	0.999 (0.996–1.0) 0.999 (0.001)	7.0 (4.0–9.8) 6.8 (1.6)
*HD-BET* (preprocessed)	False	0.942 (0.908–0.963) 0.94 (0.012)	0.893 (0.833–0.952) 0.891 (0.023)	0.999 (0.996–1.0) 0.999 (0.001)	6.2 (4.0–9.8) 6.3 (1.2)
*HD-BET-fast* (preprocessed)	True	0.938 (0.914–0.954) 0.938 (0.009)	0.892 (0.844–0.932) 0.892 (0.02)	0.998 (0.995–1.0) 0.998 (0.001)	6.6 (4.2–16.4) 6.7 (2.0)
*HD-BET-fast* (preprocessed)	False	0.947 (0.914–0.967) 0.945 (0.012)	0.904 (0.843–0.945) 0.901 (0.022)	0.999 (0.994–1.0) 0.998 (0.001)	5.6 (4.0–9.9) 5.9 (1.2)
*ROBEX* (original)	True	0.94 (0.816–0.96) 0.933 (0.029)	0.99 (0.895–1.0) 0.986 (0.017)	0.976 (0.948–0.989) 0.973 (0.01)	4.8 (4.0–16.9) 5.8 (2.7)
*ROBEX* (original)	False	0.937 (0.82–0.961) 0.934 (0.017)	0.993 (0.904–1.0) 0.989 (0.017)	0.966 (0.909–0.984) 0.964 (0.013)	4.9 (3.7–19.1) 5.7 (2.5)
*SynthStrip* (original)	True	0.95 (0.924–0.975) 0.95 (0.013)	0.915 (0.862–0.972) 0.917 (0.028)	0.998 (0.993–0.999) 0.997 (0.001)	4.7 (2.3–8.0) 4.8 (1.1)
*SynthStrip* (original)	False	0.963 (0.935–0.981) 0.962 (0.01)	0.943 (0.888–0.986) 0.942 (0.022)	0.996 (0.987–0.999) 0.996 (0.002)	4.0 (2.5–6.0) 4.1 (0.4)
*SynthStrip-noCSF* (original)	True	0.918 (0.89–0.944) 0.918 (0.012)	0.852 (0.804–0.894) 0.851 (0.021)	1.0 (0.998–1.0) 0.999 (0.0)	9.2 (5.2–20.0) 10.4 (3.4)
*SynthStrip-noCSF* (original)	False	0.94 (0.898–0.959) 0.937 (0.013)	0.888 (0.816–0.925) 0.885 (0.022)	0.999 (0.997–1.0) 0.999 (0.0)	8.0 (4.4–21.3) 8.4 (2.9)
*d-SynthStrip* (original)	True	0.938 (0.908–0.971) 0.94 (0.015)	0.887 (0.835–0.953) 0.892 (0.027)	0.999 (0.997–1.0) 0.999 (0.001)	5.1 (4.0–8.0) 5.3 (1.1)
*d-SynthStrip* (original)	False	0.96 (0.912–0.978) 0.957 (0.014)	0.932 (0.841–0.965) 0.926 (0.027)	0.998 (0.994–1.0) 0.998 (0.001)	4.0 (3.6–7.7) 4.4 (0.7)

## 4 Discussion

Rehman et al. (2020) postulate a gap between the use of skull stripping tools in research and their use in clinical routine. Robustness, computing time and user-friendly application were considered the main obstacles to overcome. We hope to narrow the implementation gap through the evaluation of different skull stripping tools in pediatric T2W MRI scans. Although not trained with a focus on this use case, all models exhibited decent median segmentation performances. Nevertheless, we recommend *SynthStrip* since it is the most robust model with a quick computational time.

In our study, *SynthStrip* achieved the highest Dice score, which measures the overall agreement between ground truth and segmentation but potentially underrepresents the contour of the brain. *SynthStrip* also had the highest HD_95_ score, which is solely based on the brain’s contour. Therefore, the model segments both the inner structures and the outer shape of the brain more reliably than the other models. Varying margins in the Bland–Altman plots indicate that *SynthStrip* handles cases well when other models’ segmentations are less satisfactory. The observed differences were statistically significant. For interpreting the results of the statistical tests, our study setting should be taken into consideration. Usually, power calculations are used to determine adequate cohort sizes for detecting an anticipated effect without involving too many subjects, which can be connected to ethical as well as economic implications (Kang, 2021). Since our study was carried out retrospectively on clinical routine data, it was not affected by these implications. Therefore, we used all available data from the clinical routine resulting in a considerably large cohort. This generally leads to more reliable results, but at the same time to statistical significance even though differences observed are small (“overpowered” setting).

The developers of *SynthStrip* (Hoopes et al., 2022) published comparisons to *ROBEX* as well as *BET* on three adult T2W MRI datasets: IXI^5^, FSM (Greve et al., 2021) and QIN (Mamonov and Kalpathy-Cramer, 2016). In the three datasets, *SynthStrip* had mean Dice scores of 0.96, 0.98 and 0.95, respectively, and outperformed *ROBEX* as well as *BET*. The two models were also outperformed by *HD-BET* in its original publication (Isensee et al., 2019) when tested on T1W scans. This is in concordance with our findings and the notion that deep learning models outperform classical skull stripping methods (Fatima et al., 2020).

In contrast to our study, Vaz et al. (2024) found that *SynthStrip* was outperformed by *HD-BET* by approximately 1% in T2W pediatric scans. Possible explanations include their small cohort and a differing age distribution (22 premature neonates). The finding of Kelley et al. (2024), that *d-SynthStrip* outperforms *SynthStrip* on pediatric T2W MRI scans, is also not supported by our results. Kelley et al. investigated the models’ performances on two pediatric cohorts with T2W scans, which led to median Dice scores of 0.97 and 0.96 for *SynthStrip*, whereas *d-SynthStrip* showed median Dice scores of approximately 0.98 in both cohorts. Although the performance of *SynthStrip* in these cohorts aligns with our measurements, the median Dice score of *d-SynthStrip* is 0.02 lower in our setting. The most likely explanation is the exclusion of CSF by Kelley et al., whereas in our study CSF was included. This explanation is supported by the segmentation performance difference between *SynthStrip* and *SynthStrip-noCSF* in our research which was also 0.02. Generally, the lack of fixed segmentation rules is an obstacle to standardized skull stripping tools. Using *SynthStrip*, the inclusion and exclusion of CSF can easily be switched through the noCSF-parameter.

The fact that *SynthStrip* can compete with the pediatric model *d-SynthStrip*, shows that its data augmentation strategy compensates for the difference in training data. *d-SynthStrip* was trained on a mixture of 57 T1W and T2W scans of children under the age of 4.5 years, whereas the training data of *SynthStrip* exclusively included T1W scans and only 10 out of 80 scans belonged to infants which were all under the age of 1.5 years. The success of the data augmentation strategy is also evident in the comparison of *SynthStrip* and *HD-BET*, which both have U-Net-based architectures. *HD-BET* was trained on as many as 6,586 MRI sequences with a fifth of them being T2-weighted but did not yield better results than *SynthStrip*.

The robustness of *SynthStrip*, reflected by its narrow value range, has also been observed in T1W pediatric MRI scans. Das et al. (2023) observed that *SynthStrip* provided good results where other models (*BET*, Freesurfer) failed. However, their finding that *SynthStrip* leaves the frontal lobe and other brain structures in T1-weighted scans intact, differs from our experience with T2W scans. Despite its high overall performance, *SynthStrip* has shortcomings. We found that parts of the upper brain slices, the temporal poles and the *Cerebellum* were prone to be omitted, which may be linked to the partial volume effect (PVE) at their borders. The PVE describes the phenomenon of capturing multiple tissues within a voxel and is known to impair the accuracy of brain segmentations (Tohka, 2014). Eventually, the PVE is also the reason why the petrous bone has, on the other hand, often been included. Albeit not being included in the brain regions *SynthStrip* was trained on, the C*hiasma opticum/*pituitary gland as well as the *Sinus sagittalis superior* were segmented in about one third of the cases. But this rate is not sufficient for using the tool in diagnostics linked to these structures (e.g., adenoma of the pituitary gland or thrombosis of the *Sinus sagittalis superior*). In alignment with the research by Vaz et al. (2024), *SynthStrip* performed best in the middle slices of the brain. Nevertheless, we witnessed errors in the skull exclusion and CSF inclusion in these slices. Due to the small size of the affected area, this did not lead to a considerable Dice score decrease.

Generally, the *SynthStrip*-related models are the computationally most efficient models. Although *BET* is faster, it requires time-consuming data preprocessing, which nullifies its advantage. In contrast, *SynthStrip*-related models can be directly applied to data from the clinical routine. The reason why Vaz et al. (2024) measured a computation time of *SynthStrip* almost 40 times above ours, is the usage of superseded consumer grade hardware for the calculations (a laptop from 2015).

For ML applications that require skull stripped MRI scans with standardized input dimensions, scans need to be resampled. In these cases, we recommend skull stripping before resampling to obtain the best possible brain segmentation. Although, we did not analyze the segmentation performance before and after resampling on the same scans, the comparison of the 162 non-resampled scans to the 37 resampled ones showed a drop in performance. Drai et al. (2022) did not recognize any age-dependent alterations in the performance of *HD-BET* or *BET*. For the latter, there was a steady performance decrease in correlation with the infants’ age in our study, whereas the performance of *SynthStrip* as well as *d-SynthStrip* increased. To our knowledge, there is no literature investigating the impact of age on the performance of *SynthStrip or d-SynthStrip*. Still, the influence could be derived from the drastic brain changes taking place within the first 6 years of life. During this period, the brain volume expands and changes in voxel intensity occur due to the process of myelination. In the younger, less myelinated brains, the contrast is lower making it more difficult to distinguish different anatomical structures from one another (Phan et al., 2018).

A limitation of our study is its monocentric design, which could cause selection bias. Further, the dataset used is skewed toward younger children with ages under 1 year. Since *SynthStrip* had its lowest performance in that age group and most of the other models did not show age-related performance alterations, this should be negligible regarding model comparisons. The fact that there are only subjects with non-pathological scans in the cohort is also a limitation. Therefore, the skull stripping performance when bleedings, cancer, or other pathologies are present, could not be assessed. Our assessment of regional segmentation performances is based on splits derived from the dimensions of the ground truth masks. Although, the assessment is therefore limited to brought areas of the brain, it aligns well with the manual findings. For directly linking segmentations performances to distinct anatomical structures extensive labeling would be required.

On the other hand, our study has several strengths. The cohort size (*n* = 199) is comparably large in the field of brain segmentation, where small sample sizes are frequent. Vaz et al. (2024) with a cohort of *n* = 22 cited multiple publications with test datasets of an equal or smaller size (Shattuck et al., 2001; Péporté et al., 2011; Eskildsen et al., 2012; Mahapatra, 2012; Serag et al., 2016; Kobashi and Udupa, 2013). According to them, the time-consuming manual segmentation is a reason for the small cohorts. In our study, we invested considerable resources in creating ground truth masks, which were quality-checked by a senior expert in the field.

In this study, we evaluated different skull stripping tools in pediatric T2W MRI scans. Generally, all tools lead to acceptable results in most cases, although some require prior data preprocessing. However, we recommend *SynthStrip* and its application prior to any resampling, if needed. *SynthStrip* showed the most stable segmentation performance on non-preprocessed (“original”) data and a small computation time, which are two requirements formulated by Rehman et al. (2020) for skull stripping tools. The third requirement, a user-friendly application, is also met with only a single line command necessary to execute the model. Especially for clinicians, it would be even more user-friendly, if a graphical user interface, such as an extension to 3D Slicer (Fedorov et al., 2012), was available. In the future, brain extraction assessment should be expanded to multicenter settings, also including pathological cases. Furthermore, future research should address the partial volume effect, as it has led to segmentation inaccuracies.

## Data Availability

The data analyzed in this study is subject to the following licenses/restrictions: a publication of pediatric MRI scans is not possible due to privacy concerns. Requests to access these datasets should be directed to Bueltmann.Eva@mh-hannover.de.

## Footnotes

1 *BET*: https://fsl.fmrib.ox.ac.uk/fsl/docs/structural/bet.html, last access November 12, 2025.

2 *ROBEX*: https://www.nitrc.org/projects/robex, last access November 12, 2025.

3 *HD-BET* & *HD-BET-fast*: https://github.com/MIC-DKFZ/HD-BET, last access November 12, 2025.

4 *SynthStrip*, *SynthStrip-noCSF* and *d-SynthStrip*: https://surfer.nmr.mgh.harvard.edu/docs/synthstrip/, last access November 12, 2025.

5 IXI-Dataset: https://brain-development.org/ixi-dataset/, last accessed August 25, 2025.
