# Supplementary material for: Skull stripping tools in pediatric T2-weighted MRI scans: a retrospective evaluation of segmentation performance
Source: Front Neurosci. 2025 Dec 18;19:1715514. doi: 10.3389/fnins.2025.1715514 (PMC12756446; doi:10.3389/fnins.2025.1715514)
Supplement: Supplementary file 1 [file Data_Sheet_1.pdf]

Supplementary Material

1 Supplementary Figures

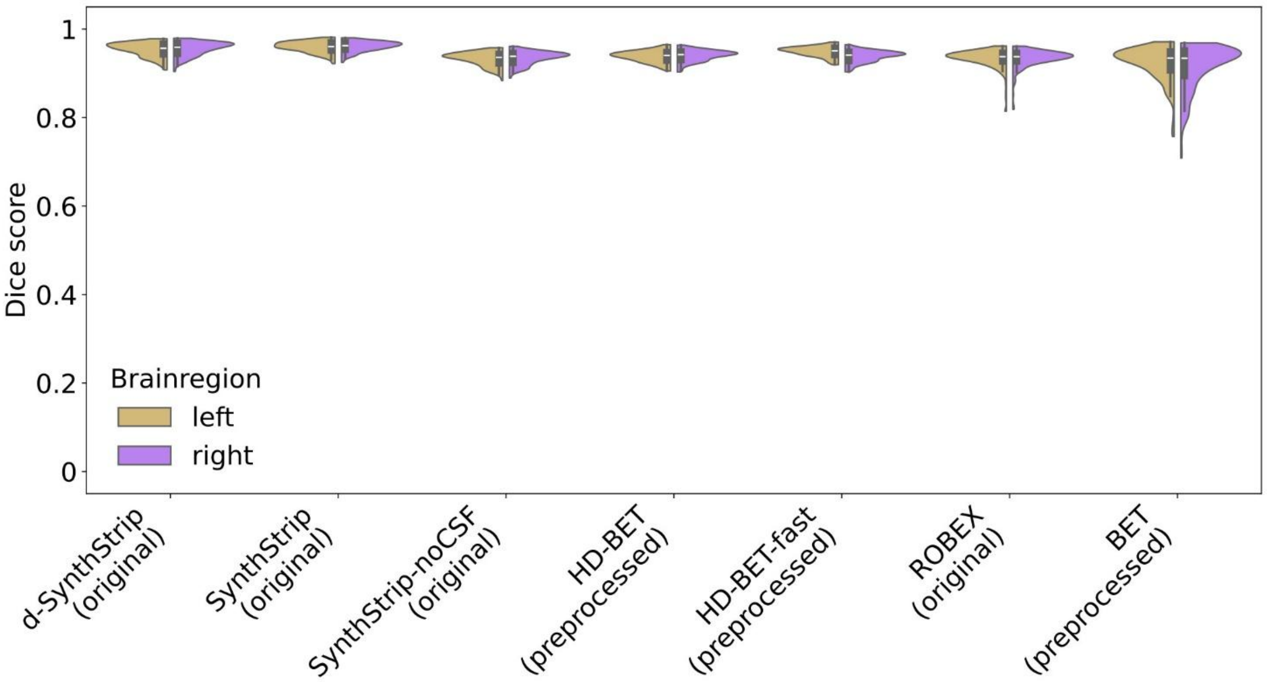

Supplementary Figure E: Regional segmentation performances (Dice score) along the transversal scan axis of all 199 cases.

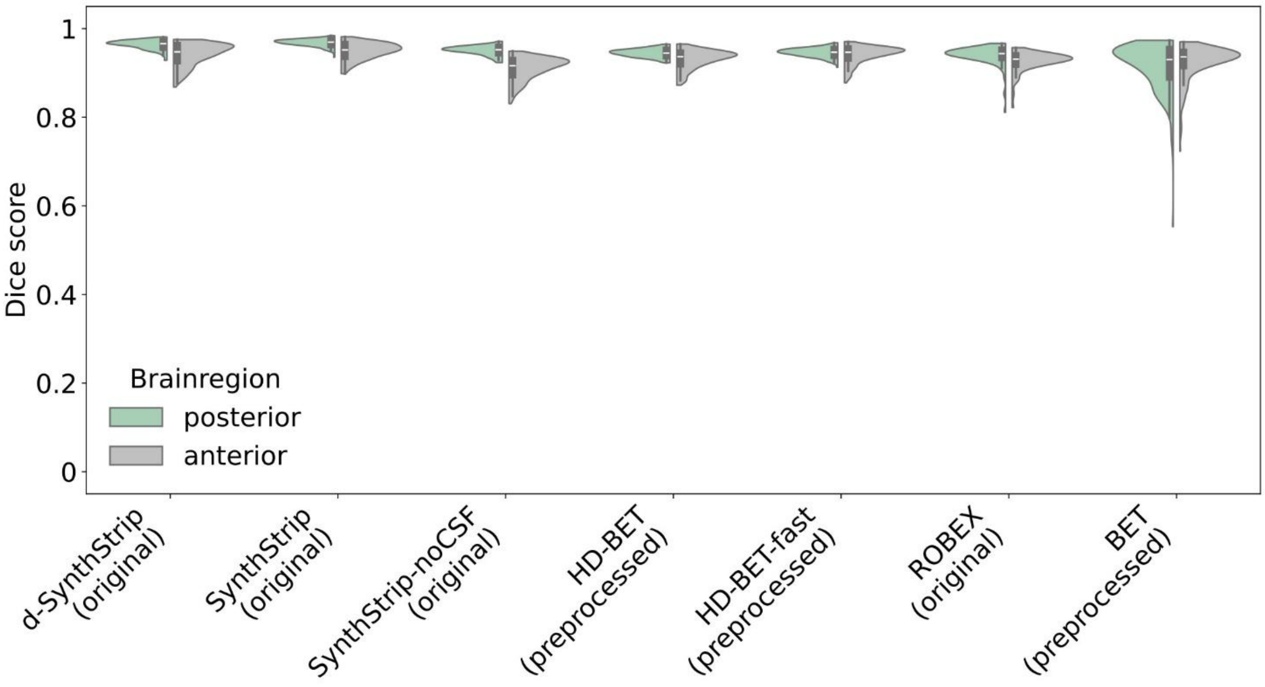

Supplementary Figure F: Regional segmentation performances (Dice score) along the sagittal scan axis of all 199 cases.

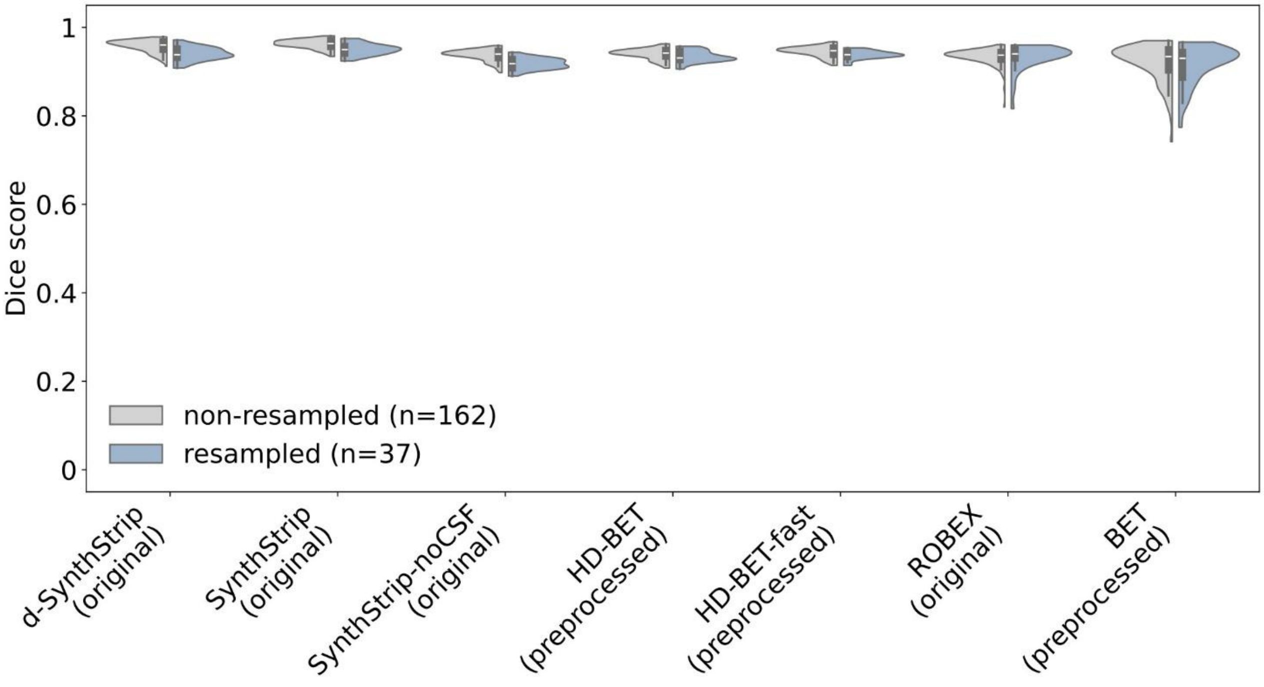

Supplementary Figure G: Segmentation performances (Dice score) on resampled and non-resampled MRI scans.

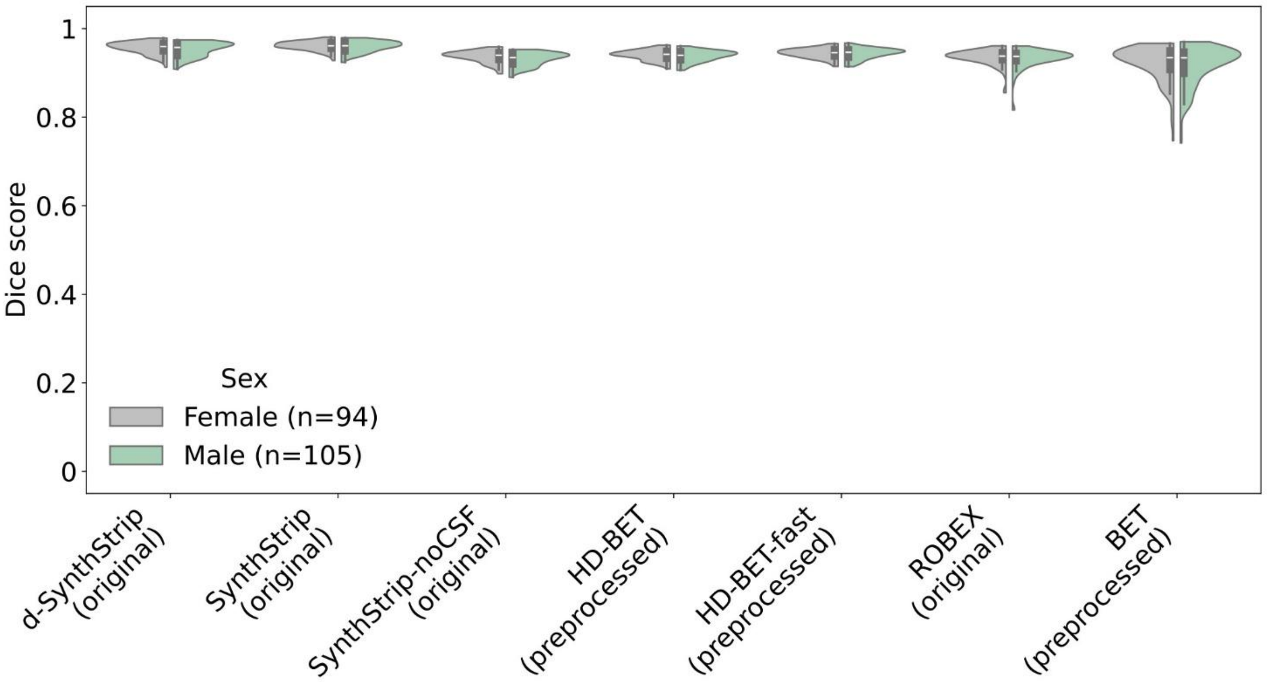

Supplementary Figure H: Overall segmentation performances (Dice score) of different models in dependence on the infants' sex.

2 Supplementary Tables

Supplementary Table A: Voxel sizes and spacing between slices of all 199 scans

Voxel Size	Spacing Between Slices	Number of Scans
0.43 mm × 0.43 mm × 4.00 mm	4.00 mm	162
0.36 mm × 0.36 mm × 5.50 mm	6.05 mm	18
0.50 mm × 0.50 mm × 3.00 mm	3.30 mm	12
0.36 mm × 0.36 mm × 4.00 mm	4.00 mm	3
0.36 mm × 0.36 mm × 4.00 mm	4.40 mm	2
0.39 mm × 0.39 mm × 4.00 mm	4.00 mm	1
0.94 mm × 0.94 mm × 5.50 mm	6.05 mm	1

Supplementary Table B: Segmentation performance metrics of each skull stripping model in combination with both the original and the preprocessed scans of all 199 cases. HD95: 95th percentile Hausdorff distance in mm.

**Model** **(Dataset)**	**Dice score** Median (Min-Max) M (SD)	**Sensitivity** Median (Min-Max) M (SD)	**Specificity** Median (Min-Max) M (SD)	**HD_{95}** Median (Min-Max) M (SD)
BET (original)	0.896 (0.664 - 0.952) 0.88 (0.057)	0.882 (0.508 - 0.999) 0.862 (0.117)	0.979 (0.96 - 0.993) 0.978 (0.007)	16.0 (4.0 - 44.6) 14.6 (7.7)
BET (preprocessed)	0.934 (0.742 - 0.97) 0.919 (0.043)	0.942 (0.635 - 0.99) 0.911 (0.078)	0.982 (0.933 - 0.997) 0.98 (0.009)	12.3 (4.0 - 44.4) 13.9 (7.9)
HD-BET (original)	0.944 (0.0 - 0.975) 0.854 (0.208)	0.906 (0.0 - 0.992) 0.8 (0.237)	0.996 (0.99 - 1.0) 0.996 (0.002)	5.7 (2.1 - 176.0) 18.3 (26.8)
HD-BET (preprocessed)	0.941 (0.906 - 0.963) 0.939 (0.012)	0.892 (0.829 - 0.952) 0.889 (0.024)	0.999 (0.996 - 1.0) 0.999 (0.001)	6.3 (4.0 - 9.8) 6.4 (1.3)
HD-BET-fast (original)	0.943 (0.643 - 0.969) 0.918 (0.065)	0.912 (0.534 - 0.983) 0.882 (0.081)	0.995 (0.912 - 0.999) 0.99 (0.016)	7.7 (4.0 - 73.0) 14.0 (14.8)
HD-BET-fast (preprocessed)	0.946 (0.914 - 0.967) 0.944 (0.012)	0.903 (0.843 - 0.945) 0.899 (0.022)	0.999 (0.994 - 1.0) 0.998 (0.001)	5.8 (4.0 - 16.4) 6.0 (1.4)
ROBEX (original)	0.938 (0.816 - 0.961) 0.934 (0.02)	0.993 (0.895 - 1.0) 0.988 (0.017)	0.967 (0.909 - 0.989) 0.966 (0.013)	4.9 (3.7 - 19.1) 5.7 (2.5)
ROBEX (preprocessed)	0.949 (0.547 - 0.964) 0.94 (0.034)	0.97 (0.378 - 0.998) 0.953 (0.055)	0.98 (0.926 - 0.999) 0.979 (0.01)	5.3 (4.0 - 34.7) 7.9 (5.8)
SynthStrip (original)	0.961 (0.924 - 0.981) 0.96 (0.012)	0.938 (0.862 - 0.986) 0.937 (0.025)	0.997 (0.987 - 0.999) 0.996 (0.002)	4.0 (2.3 - 8.0) 4.2 (0.6)
SynthStrip (preprocessed)	0.931 (0.865 - 0.967) 0.929 (0.014)	0.873 (0.762 - 0.954) 0.872 (0.026)	0.999 (0.993 - 1.0) 0.999 (0.001)	6.9 (4.0 - 20.5) 6.9 (1.6)

SynthStrip-noCSF (original)	0.938 (0.89 - 0.959)<br>0.933 (0.015)	0.885 (0.804 - 0.925)<br>0.878 (0.025)	0.999 (0.997 - 1.0)<br>0.999 (0.0)	8.0 (4.4 - 21.3)<br>8.7 (3.1)
SynthStrip-noCSF (preprocessed)	0.884 (0.82 - 0.928)<br>0.883 (0.014)	0.793 (0.695 - 0.867)<br>0.791 (0.022)	1.0 (0.999 - 1.0)<br>1.0 (0.0)	16.0 (8.1 - 32.4)<br>16.6 (5.3)
d-SynthStrip (original)	0.958 (0.908 - 0.978)<br>0.954 (0.015)	0.925 (0.835 - 0.965)<br>0.92 (0.03)	0.998 (0.994 - 1.0)<br>0.998 (0.001)	4.0 (3.6 - 8.0)<br>4.5 (0.9)
d-SynthStrip (preprocessed)	0.93 (0.877 - 0.965)<br>0.927 (0.018)	0.871 (0.781 - 0.938)<br>0.866 (0.033)	1.0 (0.997 - 1.0)<br>1.0 (0.0)	7.0 (4.2 - 10.9)<br>7.0 (1.3)

Supplementary Table C: Two-sided Wilcoxon signed-rank test with *p*-values and adjusted *p*-values using Bonferroni correction. The significance level was set to 0.05. Asterisk (*) indicates statistical significance.

**Model comparison**	**Metric**	**Median difference**	***p*-value**	**adjusted *p*-value**
d-SynthStrip (original) vs. SynthStrip (original)	Dice score	- 0.003	< 0.001	< 0.001*
	HD_{95}	< 0.1 mm	< 0.001	< 0.001*
d-SynthStrip (original) vs. SynthStrip-noCSF (original)	Dice score	0.020	< 0.001	< 0.001*
	HD_{95}	-4.0 mm	< 0.001	< 0.001*
d-SynthStrip (original) vs. HD-BET (preprocessed)	Dice score	0.017	< 0.001	< 0.001*
	HD_{95}	-2.2 mm	< 0.001	< 0.001*
d-SynthStrip (original) vs. HD-BET-fast (preprocessed)	Dice score	0.012	< 0.001	< 0.001*
	HD_{95}	-1.8 mm	< 0.001	< 0.001*
d-SynthStrip (original) vs. ROBEX (original)	Dice score	0.020	< 0.001	< 0.001*
	HD_{95}	-0.9 mm	< 0.001	< 0.001*
d-SynthStrip (original) vs. BET (preprocessed)	Dice score	0.024	< 0.001	< 0.001*

	HD_{95}	-8.3 mm	< 0.001	< 0.001*
SynthStrip (original) vs. SynthStrip-noCSF (original)	Dice score	0.023	< 0.001	< 0.001*
	HD_{95}	-4.0 mm	< 0.001	< 0.001*
SynthStrip (original) vs. HD-BET (preprocessed)	Dice score	0.020	< 0.001	< 0.001*
	HD_{95}	-2.3 mm	< 0.001	< 0.001*
SynthStrip (original) vs. HD-BET-fast (preprocessed)	Dice score	0.015	< 0.001	< 0.001*
	HD_{95}	-1.8 mm	< 0.001	< 0.001*
SynthStrip (original) vs. ROBEX (original)	Dice score	0.023	< 0.001	< 0.001*
	HD_{95}	-0.9 mm	< 0.001	< 0.001*
SynthStrip (original) vs. BET (preprocessed)	Dice score	0.027	< 0.001	< 0.001*
	HD_{95}	-8.3 mm	< 0.001	< 0.001*
SynthStrip-noCSF (original) vs. HD-BET (preprocessed)	Dice score	- 0.003	< 0.001	< 0.001*
	HD_{95}	1.7 mm	< 0.001	< 0.001*
SynthStrip-noCSF (original) vs. HD-BET-fast (preprocessed)	Dice score	- 0.008	< 0.001	< 0.001*
	HD_{95}	2.2 mm	< 0.001	< 0.001*
SynthStrip-noCSF (original) vs. ROBEX (original)	Dice score	< 0.001	0.465	1
	HD_{95}	3.1 mm	< 0.001	< 0.001*
SynthStrip-noCSF (original) vs. BET (preprocessed)	Dice score	0.004	0.008	0.340
	HD_{95}	-4.3 mm	< 0.001	< 0.001*
HD-BET (preprocessed) vs. HD-BET-fast (preprocessed)	Dice score	- 0.005	< 0.001	< 0.001*
	HD_{95}	0.4 mm	< 0.001	< 0.001*
HD-BET (preprocessed) vs. ROBEX (original)	Dice score	0.003	0.051	1
	HD_{95}	1.4 mm	< 0.001	< 0.001*

HD-BET (preprocessed) vs. BET (preprocessed)	Dice score	0.007	< 0.01	< 0.05*
	HD_{95}	-6.0 mm	< 0.001	< 0.001*
HD-BET-fast (preprocessed) vs. ROBEX (original)	Dice score	0.008	< 0.01	< 0.001*
	HD_{95}	0,9 mm	< 0.01	< 0.05*
HD-BET-fast (preprocessed) vs. BET (preprocessed)	Dice score	0.012	< 0.01	< 0.001*
	HD_{95}	-6,5 mm	< 0.001	< 0.001*
ROBEX (original) vs BET (preprocessed)	Dice score	0.004	< 0.01	< 0.05*
	HD_{95}	-7,4 mm	< 0.001	< 0.001*

Supplementary Table D: Regional segmentation performance metrics along the longitudinal scan axis of all 199 cases. HD95: 95th percentile Hausdorff distance in mm.

**Model (Dataset)**	**Brain Region**	**Dice score** Median (Min-Max) M (SD)	**Sensitivity** Median (Min-Max) M (SD)	**Specificity** Median (Min-Max) M (SD)	**HD_{95}** Median (Min-Max) M (SD)
BET (preprocessed)	central	0.964 (0.792 - 0.993) 0.953 (0.036)	0.952 (0.66 - 0.998) 0.933 (0.067)	0.989 (0.974 - 0.998) 0.989 (0.005)	8.0 (0.4 - 29.1) 8.4 (5.6)
BET (preprocessed)	inferior	0.909 (0.414 - 0.95) 0.89 (0.075)	0.931 (0.266 - 0.97) 0.913 (0.101)	0.982 (0.853 - 0.999) 0.977 (0.019)	11.2 (4.0 - 51.2) 13.0 (7.6)
BET (preprocessed)	superior	0.916 (0.533 - 0.968) 0.883 (0.083)	0.95 (0.377 - 1.0) 0.879 (0.151)	0.978 (0.935 - 0.999) 0.977 (0.013)	6.4 (3.4 - 32.5) 10.0 (6.8)
HD-BET (preprocessed)	central	0.98 (0.958 - 0.988) 0.979 (0.006)	0.964 (0.92 - 0.984) 0.962 (0.012)	0.999 (0.995 - 1.0) 0.999 (0.001)	2.3 (1.2 - 6.2) 2.6 (0.9)
HD-BET (preprocessed)	inferior	0.921 (0.891 - 0.956) 0.921 (0.013)	0.863 (0.806 - 0.93) 0.863 (0.025)	0.999 (0.993 - 1.0) 0.998 (0.001)	4.7 (4.0 - 6.3) 4.8 (0.6)
HD-BET (preprocessed)	superior	0.887 (0.789 - 0.951) 0.886 (0.031)	0.799 (0.652 - 0.952) 0.799 (0.054)	1.0 (0.993 - 1.0) 0.999 (0.001)	6.9 (4.0 - 11.4) 7.0 (1.5)

HD-BET-fast (preprocessed)	central	0.979 (0.954 - 0.987)<br>0.977 (0.006)	0.962 (0.914 - 0.982)<br>0.959 (0.012)	0.999 (0.995 - 1.0)<br>0.998 (0.001)	2.6 (1.3 - 10.0)<br>2.8 (1.1)
HD-BET-fast (preprocessed)	inferior	0.916 (0.873 - 0.956)<br>0.916 (0.014)	0.852 (0.786 - 0.926)<br>0.854 (0.027)	0.999 (0.992 - 1.0)<br>0.998 (0.001)	4.9 (4.0 - 10.0)<br>5.1 (0.9)
HD-BET-fast (preprocessed)	superior	0.915 (0.821 - 0.962)<br>0.91 (0.025)	0.85 (0.698 - 0.929)<br>0.842 (0.044)	0.999 (0.987 - 1.0)<br>0.998 (0.002)	6.3 (4.0 - 25.1)<br>6.6 (1.9)
ROBEX (original)	central	0.961 (0.878 - 0.976)<br>0.957 (0.014)	0.998 (0.847 - 1.0)<br>0.995 (0.017)	0.961 (0.902 - 0.984)<br>0.959 (0.013)	4.0 (2.6 - 21.7)<br>4.5 (2.0)
ROBEX (original)	inferior	0.925 (0.658 - 0.955)<br>0.912 (0.047)	0.974 (0.61 - 1.0)<br>0.962 (0.059)	0.983 (0.915 - 0.997)<br>0.979 (0.013)	4.4 (2.9 - 22.2)<br>6.1 (3.5)
ROBEX (original)	superior	0.917 (0.814 - 0.963)<br>0.915 (0.026)	0.999 (0.942 - 1.0)<br>0.996 (0.007)	0.959 (0.881 - 0.992)<br>0.956 (0.02)	4.6 (2.9 - 12.0)<br>4.9 (1.1)
SynthStrip (original)	central	0.985 (0.962 - 0.993)<br>0.984 (0.006)	0.982 (0.945 - 0.996)<br>0.98 (0.01)	0.995 (0.982 - 0.998)<br>0.995 (0.002)	1.4 (0.4 - 4.1)<br>1.6 (0.7)
SynthStrip (original)	inferior	0.947 (0.9 - 0.97)<br>0.945 (0.013)	0.929 (0.827 - 0.984)<br>0.927 (0.034)	0.996 (0.981 - 1.0)<br>0.995 (0.004)	4.0 (2.1 - 6.3)<br>3.9 (0.6)
SynthStrip (original)	superior	0.935 (0.84 - 0.981)<br>0.932 (0.027)	0.882 (0.725 - 0.983)<br>0.879 (0.05)	0.999 (0.992 - 1.0)<br>0.999 (0.002)	4.0 (1.5 - 8.0)<br>4.3 (1.0)
SynthStrip-noCSF (original)	central	0.97 (0.93 - 0.983)<br>0.968 (0.009)	0.945 (0.883 - 0.97)<br>0.941 (0.015)	0.999 (0.994 - 1.0)<br>0.999 (0.001)	4.0 (1.8 - 12.0)<br>4.7 (2.1)
SynthStrip-noCSF (original)	inferior	0.909 (0.812 - 0.939)<br>0.902 (0.025)	0.839 (0.685 - 0.894)<br>0.827 (0.042)	0.999 (0.997 - 1.0)<br>0.999 (0.001)	5.2 (4.0 - 12.6)<br>5.4 (1.3)
SynthStrip-noCSF (original)	superior	0.904 (0.812 - 0.949)<br>0.899 (0.028)	0.825 (0.684 - 0.913)<br>0.819 (0.046)	1.0 (0.998 - 1.0)<br>1.0 (0.0)	6.1 (4.0 - 16.0)<br>6.8 (2.5)
d-SynthStrip (original)	central	0.983 (0.95 - 0.991)<br>0.981 (0.008)	0.97 (0.906 - 0.989)<br>0.966 (0.017)	0.998 (0.991 - 1.0)<br>0.998 (0.001)	1.8 (0.9 - 5.3)<br>2.1 (1.0)

d-SynthStrip (original)	inferior	0.932 (0.865 - 0.961)<br>0.927 (0.019)	0.892 (0.767 - 0.95)<br>0.882 (0.038)	0.997 (0.991 - 1.0)<br>0.997 (0.002)	4.1 (3.5 - 7.5)<br>4.4 (0.7)
d-SynthStrip (original)	superior	0.939 (0.833 - 0.98)<br>0.931 (0.031)	0.887 (0.713 - 0.97)<br>0.875 (0.056)	1.0 (0.991 - 1.0)<br>0.999 (0.001)	4.0 (2.1 - 8.0)<br>4.6 (1.2)

Supplementary Table E: Regional segmentation performance metrics along the transversal scan axis of all 199 cases. HD95: 95th percentile Hausdorff distance in mm.

**Model (Dataset)**	**Brain Region**	**Dice score** Median (Min-Max) M (SD)	**Sensitivity** Median (Min-Max) M (SD)	**Specificity** Median (Min-Max) M (SD)	**HD_{95}** Median (Min-Max) M (SD)
BET (preprocessed)	left	0.934 (0.757 - 0.971)<br>0.922 (0.038)	0.946 (0.636 - 0.989)<br>0.92 (0.069)	0.981 (0.93 - 0.996)<br>0.979 (0.01)	10.3 (4.0 - 42.0)<br>12.1 (7.0)
BET (preprocessed)	right	0.934 (0.709 - 0.969)<br>0.915 (0.05)	0.941 (0.609 - 0.992)<br>0.903 (0.091)	0.983 (0.935 - 0.997)<br>0.982 (0.009)	10.7 (3.7 - 43.0)<br>12.6 (7.9)
HD-BET (preprocessed)	left	0.94 (0.905 - 0.964)<br>0.938 (0.013)	0.891 (0.827 - 0.959)<br>0.888 (0.025)	0.999 (0.995 - 1.0)<br>0.999 (0.001)	6.0 (3.5 - 9.5)<br>6.1 (1.3)
HD-BET (preprocessed)	right	0.942 (0.903 - 0.964)<br>0.94 (0.012)	0.894 (0.825 - 0.953)<br>0.891 (0.024)	0.999 (0.996 - 1.0)<br>0.999 (0.001)	5.7 (3.7 - 9.5)<br>6.0 (1.2)
HD-BET-fast (preprocessed)	left	0.951 (0.92 - 0.97)<br>0.949 (0.011)	0.912 (0.855 - 0.952)<br>0.909 (0.022)	0.998 (0.994 - 1.0)<br>0.998 (0.001)	4.8 (3.8 - 8.3)<br>5.1 (0.9)
HD-BET-fast (preprocessed)	right	0.941 (0.903 - 0.964)<br>0.938 (0.013)	0.893 (0.827 - 0.939)<br>0.889 (0.024)	0.999 (0.992 - 1.0)<br>0.999 (0.001)	5.8 (4.0 - 21.9)<br>6.1 (1.7)
ROBEX (original)	left	0.938 (0.814 - 0.962)<br>0.934 (0.02)	0.993 (0.892 - 1.0)<br>0.989 (0.016)	0.967 (0.906 - 0.99)<br>0.965 (0.013)	4.6 (3.0 - 16.1)<br>5.4 (2.2)
ROBEX (original)	right	0.938 (0.819 - 0.961)<br>0.934 (0.02)	0.993 (0.892 - 1.0)<br>0.988 (0.017)	0.967 (0.912 - 0.989)<br>0.966 (0.013)	4.6 (3.0 - 18.4)<br>5.4 (2.3)
SynthStrip (original)	left	0.96 (0.922 - 0.981)<br>0.959 (0.012)	0.936 (0.86 - 0.986)<br>0.935 (0.026)	0.997 (0.986 - 0.999)<br>0.996 (0.002)	4.0 (1.9 - 6.9)<br>4.0 (0.6)

SynthStrip (original)	right	0.963 (0.925 - 0.98)<br>0.961 (0.011)	0.941 (0.863 - 0.987)<br>0.939 (0.024)	0.996 (0.987 - 0.999)<br>0.996 (0.002)	4.0 (1.9 - 7.7)<br>3.9 (0.7)
SynthStrip-noCSF (original)	left	0.936 (0.884 - 0.958)<br>0.932 (0.015)	0.882 (0.798 - 0.922)<br>0.876 (0.026)	0.999 (0.997 - 1.0)<br>0.999 (0.0)	4.9 (4.0 - 10.6)<br>5.3 (1.2)
SynthStrip-noCSF (original)	right	0.938 (0.889 - 0.961)<br>0.935 (0.015)	0.886 (0.803 - 0.93)<br>0.881 (0.025)	0.999 (0.997 - 1.0)<br>0.999 (0.001)	4.6 (4.0 - 8.6)<br>5.1 (1.2)
d-SynthStrip (original)	left	0.957 (0.908 - 0.978)<br>0.953 (0.016)	0.925 (0.834 - 0.965)<br>0.918 (0.03)	0.998 (0.994 - 1.0)<br>0.998 (0.001)	4.0 (2.8 - 7.6)<br>4.3 (0.7)
d-SynthStrip (original)	right	0.959 (0.904 - 0.979)<br>0.955 (0.015)	0.929 (0.827 - 0.967)<br>0.921 (0.03)	0.998 (0.994 - 1.0)<br>0.998 (0.001)	4.0 (2.5 - 8.0)<br>4.2 (0.7)

Supplementary Table F: Regional segmentation performance metrics along the sagittal scan axis of all 199 cases. HD95: 95th percentile Hausdorff distance in mm.

**Model (Dataset)**	**Brain Region**	**Dice score**<br>Median (Min-Max)<br>M (SD)	**Sensitivity**<br>Median (Min-Max)<br>M (SD)	**Specificity**<br>Median (Min-Max)<br>M (SD)	**HD_{95}**<br>Median (Min-Max)<br>M (SD)
BET (preprocessed)	anterior	0.936 (0.723 - 0.97)<br>0.923 (0.04)	0.986 (0.639 - 0.997)<br>0.953 (0.07)	0.977 (0.895 - 0.996)<br>0.975 (0.014)	8.5 (4.0 - 36.3)<br>11.2 (6.6)
BET (preprocessed)	posterior	0.93 (0.553 - 0.973)<br>0.914 (0.055)	0.908 (0.385 - 0.989)<br>0.879 (0.096)	0.988 (0.955 - 0.998)<br>0.987 (0.006)	11.1 (4.0 - 47.7)<br>12.7 (7.9)
HD-BET (preprocessed)	anterior	0.937 (0.872 - 0.965)<br>0.931 (0.021)	0.884 (0.775 - 0.948)<br>0.877 (0.038)	0.999 (0.996 - 1.0)<br>0.999 (0.001)	5.6 (4.0 - 10.5)<br>6.1 (1.6)
HD-BET (preprocessed)	posterior	0.945 (0.923 - 0.965)<br>0.945 (0.009)	0.898 (0.858 - 0.969)<br>0.899 (0.019)	0.999 (0.994 - 1.0)<br>0.999 (0.001)	5.8 (3.4 - 9.4)<br>5.9 (1.1)
HD-BET-fast (preprocessed)	anterior	0.947 (0.878 - 0.97)<br>0.941 (0.019)	0.906 (0.784 - 0.957)<br>0.896 (0.036)	0.998 (0.995 - 1.0)<br>0.998 (0.001)	4.7 (4.0 - 9.6)<br>5.3 (1.3)

HD-BET-fast (preprocessed)	posterior	0.947 (0.913 - 0.968)<br>0.946 (0.01)	0.902 (0.843 - 0.949)<br>0.902 (0.019)	0.999 (0.99 - 1.0)<br>0.998 (0.001)	5.7 (4.0 - 21.6)<br>6.0 (1.7)
ROBEX (original)	anterior	0.931 (0.822 - 0.957)<br>0.926 (0.022)	0.996 (0.791 - 1.0)<br>0.99 (0.025)	0.971 (0.913 - 0.99)<br>0.969 (0.012)	4.4 (3.4 - 23.1)<br>5.4 (2.6)
ROBEX (original)	posterior	0.944 (0.811 - 0.967)<br>0.94 (0.021)	0.991 (0.818 - 1.0)<br>0.987 (0.022)	0.963 (0.887 - 0.989)<br>0.961 (0.016)	4.8 (3.1 - 19.9)<br>5.4 (2.3)
SynthStrip (original)	anterior	0.952 (0.898 - 0.982)<br>0.949 (0.018)	0.918 (0.817 - 0.983)<br>0.914 (0.038)	0.998 (0.989 - 1.0)<br>0.998 (0.002)	4.0 (2.1 - 8.0)<br>4.2 (0.8)
SynthStrip (original)	posterior	0.969 (0.935 - 0.984)<br>0.968 (0.008)	0.956 (0.886 - 0.99)<br>0.955 (0.018)	0.995 (0.98 - 0.999)<br>0.994 (0.003)	4.0 (1.5 - 6.0)<br>3.8 (0.6)
SynthStrip-noCSF (original)	anterior	0.916 (0.831 - 0.949)<br>0.91 (0.025)	0.847 (0.711 - 0.908)<br>0.836 (0.042)	1.0 (0.998 - 1.0)<br>1.0 (0.0)	8.0 (4.0 - 15.4)<br>8.5 (2.5)
SynthStrip-noCSF (original)	posterior	0.952 (0.923 - 0.971)<br>0.951 (0.01)	0.913 (0.859 - 0.948)<br>0.911 (0.019)	0.999 (0.995 - 1.0)<br>0.999 (0.001)	4.7 (4.0 - 13.8)<br>5.3 (1.7)
d-SynthStrip (original)	anterior	0.948 (0.868 - 0.975)<br>0.941 (0.025)	0.907 (0.769 - 0.962)<br>0.897 (0.046)	0.999 (0.995 - 1.0)<br>0.998 (0.001)	4.0 (3.0 - 8.1)<br>4.5 (1.0)
d-SynthStrip (original)	posterior	0.966 (0.929 - 0.981)<br>0.964 (0.01)	0.94 (0.87 - 0.976)<br>0.937 (0.021)	0.998 (0.992 - 1.0)<br>0.997 (0.002)	4.0 (2.5 - 7.4)<br>4.0 (0.4)

Supplementary Table G: *p*-values and adjusted *p*-values using Bonferroni correction of two-sided Brunner Munzel tests comparing the segmentation of 162 non-resampled scans with 37 resampled ones separately for each model and evaluation metric. The significance level was set to 0.05. Asterisk (*) indicates statistical significance.

Model (Dataset)	Metric	Median difference	*p*-value	adjusted *p*-value
BET (preprocessed)	Dice score	0.004	0.660	1
	HD_{95}	1.5 mm	0.949	1
HD-BET (preprocessed)	Dice score	0.011	0.042	0.586
	HD_{95}	-0.8 mm	0.080	1
HD-BET-fast (preprocessed)	Dice score	0.009	< 0.001	< 0.001*
	HD_{95}	-1.0 mm	0.004	0.062
ROBEX (original)	Dice score	-0.003	0.266	1
	HD_{95}	0.1 mm	0.882	1
SynthStrip (original)	Dice score	0.014	< 0.001	< 0.001*
	HD_{95}	-0.7 mm	< 0.001	< 0.001*
SynthStrip-noCSF (original)	Dice score	0.022	< 0.001	< 0.001*
	HD_{95}	-1.2 mm	< 0.001	< 0.001*
d-SynthStrip (original)	Dice score	0.022	< 0.001	< 0.001*
	HD_{95}	-1.1 mm	< 0.001	< 0.001*

Supplementary Table H: Segmentation performance metrics of different models in dependence on the infants' sex (female (f): n=94, male (m): n=105). HD95: 95th percentile Hausdorff distance in mm.

**Model (Dataset)**	**Sex**	**Dice score** Median (Min-Max) M (SD)	**Sensitivity** Median (Min-Max) M (SD)	**Specificity** Median (Min-Max) M (SD)	**HD_{95}** Median (Min-Max) M (SD)
BET (preprocessed)	f	0.934 (0.747 - 0.967) 0.922 (0.041)	0.94 (0.635 - 0.986) 0.913 (0.076)	0.983 (0.962 - 0.997) 0.983 (0.006)	12.2 (4.0 - 29.1) 13.2 (7.1)
BET (preprocessed)	m	0.934 (0.742 - 0.97) 0.916 (0.045)	0.943 (0.656 - 0.99) 0.909 (0.081)	0.98 (0.933 - 0.995) 0.978 (0.011)	12.5 (4.0 - 44.4) 14.5 (8.5)
HD-BET (preprocessed)	f	0.942 (0.909 - 0.963) 0.94 (0.012)	0.894 (0.834 - 0.952) 0.892 (0.024)	0.999 (0.996 - 1.0) 0.999 (0.001)	6.1 (4.0 - 9.8) 6.2 (1.3)
HD-BET (preprocessed)	m	0.94 (0.906 - 0.96) 0.938 (0.013)	0.891 (0.829 - 0.934) 0.887 (0.024)	0.999 (0.996 - 1.0) 0.999 (0.001)	6.5 (4.0 - 9.8) 6.6 (1.3)
HD-BET-fast (preprocessed)	f	0.946 (0.915 - 0.966) 0.944 (0.011)	0.901 (0.843 - 0.945) 0.9 (0.021)	0.999 (0.995 - 1.0) 0.998 (0.001)	5.6 (4.0 - 16.4) 6.0 (1.6)
HD-BET-fast (preprocessed)	m	0.946 (0.914 - 0.967) 0.943 (0.012)	0.904 (0.844 - 0.945) 0.898 (0.023)	0.998 (0.994 - 1.0) 0.998 (0.001)	5.8 (4.0 - 9.9) 6.1 (1.2)
ROBEX (original)	f	0.938 (0.855 - 0.961) 0.935 (0.017)	0.992 (0.916 - 1.0) 0.987 (0.016)	0.969 (0.927 - 0.989) 0.969 (0.011)	4.9 (3.8 - 13.1) 5.5 (1.8)
ROBEX (original)	m	0.937 (0.816 - 0.96) 0.932 (0.022)	0.994 (0.895 - 1.0) 0.989 (0.018)	0.966 (0.909 - 0.987) 0.963 (0.013)	4.9 (3.7 - 19.1) 6.0 (2.9)
SynthStrip (original)	f	0.961 (0.928 - 0.981) 0.961 (0.01)	0.941 (0.87 - 0.986) 0.939 (0.023)	0.997 (0.99 - 0.999) 0.996 (0.002)	4.0 (2.3 - 7.4) 4.1 (0.6)
SynthStrip (original)	m	0.961 (0.924 - 0.979) 0.959 (0.012)	0.937 (0.862 - 0.982) 0.936 (0.027)	0.996 (0.987 - 0.999) 0.996 (0.002)	4.0 (3.0 - 8.0) 4.3 (0.7)
SynthStrip-noCSF (original)	f	0.94 (0.898 - 0.959) 0.936 (0.014)	0.889 (0.816 - 0.925) 0.884 (0.024)	0.999 (0.998 - 1.0) 0.999 (0.0)	7.2 (4.4 - 17.5) 7.7 (2.6)

SynthStrip-noCSF (original)	m	0.934 (0.89 - 0.953)<br>0.931 (0.015)	0.882 (0.804 - 0.915)<br>0.874 (0.026)	0.999 (0.997 - 1.0)<br>0.999 (0.001)	8.3 (5.1 - 21.3)<br>9.6 (3.3)
d-SynthStrip (original)	f	0.959 (0.912 - 0.978)<br>0.956 (0.014)	0.929 (0.841 - 0.965)<br>0.924 (0.027)	0.998 (0.994 - 1.0)<br>0.998 (0.001)	4.0 (4.0 - 7.0)<br>4.3 (0.7)
d-SynthStrip (original)	m	0.957 (0.908 - 0.974)<br>0.952 (0.017)	0.923 (0.835 - 0.963)<br>0.916 (0.032)	0.998 (0.994 - 1.0)<br>0.998 (0.001)	4.2 (3.6 - 8.0)<br>4.7 (1.0)

Supplementary Table J: Segmentation performance metrics of different models in dependence on the infants' ages in years (1 year: n=75, 2 years: n=47, 3 years: n=34, 4 years: n=16, 5 years: n=27).

**Model (Dataset)**	**Age [Years]**	**Dice score** Median (Min-Max) M (SD)	**Sensitivity** Median (Min-Max) M (SD)	**Specificity** Median (Min-Max) M (SD)	**HD_{95}** Median (Min-Max) M (SD)
BET (preprocessed)	1	0.95 (0.872 - 0.97)<br>0.947 (0.017)	0.974 (0.837 - 0.987)<br>0.967 (0.025)	0.983 (0.971 - 0.997)<br>0.983 (0.006)	6.0 (4.0 - 28.0)<br>7.2 (4.1)
BET (preprocessed)	2	0.937 (0.876 - 0.964)<br>0.933 (0.02)	0.941 (0.816 - 0.977)<br>0.932 (0.036)	0.982 (0.959 - 0.989)<br>0.98 (0.006)	12.3 (5.6 - 23.6)<br>13.2 (4.7)
BET (preprocessed)	3	0.911 (0.821 - 0.944)<br>0.903 (0.03)	0.892 (0.764 - 0.99)<br>0.877 (0.058)	0.982 (0.95 - 0.995)<br>0.98 (0.01)	18.8 (9.2 - 34.8)<br>19.1 (5.3)
BET (preprocessed)	4	0.883 (0.802 - 0.945)<br>0.885 (0.033)	0.838 (0.692 - 0.943)<br>0.848 (0.068)	0.98 (0.933 - 0.992)<br>0.976 (0.017)	22.6 (14.6 - 31.0)<br>21.8 (4.4)
BET (preprocessed)	5	0.857 (0.742 - 0.946)<br>0.856 (0.055)	0.777 (0.635 - 0.956)<br>0.801 (0.097)	0.98 (0.94 - 0.992)<br>0.978 (0.011)	23.6 (9.5 - 44.4)<br>22.9 (7.1)
HD-BET (preprocessed)	1	0.932 (0.906 - 0.96)<br>0.932 (0.014)	0.877 (0.829 - 0.952)<br>0.879 (0.029)	0.999 (0.996 - 1.0)<br>0.999 (0.001)	6.7 (4.0 - 9.8)<br>6.6 (1.6)
HD-BET (preprocessed)	2	0.94 (0.917 - 0.957)<br>0.938 (0.01)	0.888 (0.847 - 0.921)<br>0.887 (0.018)	0.999 (0.997 - 1.0)<br>0.999 (0.001)	6.2 (4.7 - 9.3)<br>6.5 (1.1)
HD-BET (preprocessed)	3	0.945 (0.927 - 0.961)<br>0.945 (0.008)	0.9 (0.867 - 0.932)<br>0.9 (0.015)	0.999 (0.996 - 0.999)<br>0.999 (0.001)	6.0 (4.2 - 8.0)<br>6.1 (0.8)

HD-BET (preprocessed)	4	0.948 (0.927 - 0.96)<br>0.947 (0.009)	0.903 (0.871 - 0.931)<br>0.902 (0.016)	0.999 (0.997 - 1.0)<br>0.999 (0.001)	5.9 (4.2 - 9.3)<br>6.2 (1.3)
HD-BET (preprocessed)	5	0.944 (0.93 - 0.963)<br>0.946 (0.009)	0.899 (0.871 - 0.936)<br>0.901 (0.017)	0.999 (0.996 - 1.0)<br>0.998 (0.001)	6.1 (4.1 - 9.0)<br>6.1 (1.1)
HD-BET-fast (preprocessed)	1	0.936 (0.914 - 0.954)<br>0.935 (0.011)	0.885 (0.843 - 0.932)<br>0.884 (0.021)	0.999 (0.995 - 1.0)<br>0.999 (0.001)	6.4 (4.0 - 16.4)<br>6.5 (1.7)
HD-BET-fast (preprocessed)	2	0.946 (0.922 - 0.956)<br>0.944 (0.008)	0.903 (0.857 - 0.921)<br>0.899 (0.015)	0.999 (0.997 - 1.0)<br>0.998 (0.001)	5.9 (5.0 - 9.9)<br>6.2 (1.0)
HD-BET-fast (preprocessed)	3	0.953 (0.935 - 0.966)<br>0.952 (0.007)	0.918 (0.882 - 0.945)<br>0.914 (0.014)	0.998 (0.994 - 0.999)<br>0.998 (0.001)	5.4 (4.1 - 9.5)<br>5.6 (1.0)
HD-BET-fast (preprocessed)	4	0.954 (0.936 - 0.967)<br>0.954 (0.008)	0.917 (0.887 - 0.941)<br>0.917 (0.014)	0.998 (0.996 - 0.999)<br>0.998 (0.001)	5.2 (4.0 - 8.3)<br>5.4 (1.0)
HD-BET-fast (preprocessed)	5	0.951 (0.929 - 0.966)<br>0.952 (0.008)	0.914 (0.872 - 0.945)<br>0.914 (0.016)	0.998 (0.995 - 0.999)<br>0.998 (0.001)	5.2 (4.0 - 9.5)<br>5.4 (1.2)
ROBEX (original)	1	0.936 (0.816 - 0.956)<br>0.929 (0.026)	0.994 (0.921 - 1.0)<br>0.991 (0.013)	0.971 (0.929 - 0.987)<br>0.971 (0.01)	4.7 (4.0 - 15.5)<br>5.6 (2.5)
ROBEX (original)	2	0.943 (0.911 - 0.961)<br>0.941 (0.012)	0.99 (0.916 - 0.997)<br>0.984 (0.019)	0.971 (0.938 - 0.989)<br>0.969 (0.011)	4.6 (3.7 - 13.1)<br>5.5 (2.2)
ROBEX (original)	3	0.938 (0.907 - 0.959)<br>0.938 (0.011)	0.994 (0.974 - 0.998)<br>0.993 (0.005)	0.962 (0.936 - 0.982)<br>0.961 (0.011)	4.9 (3.8 - 11.1)<br>5.3 (1.4)
ROBEX (original)	4	0.935 (0.9 - 0.955)<br>0.932 (0.017)	0.992 (0.915 - 0.998)<br>0.986 (0.02)	0.96 (0.909 - 0.979)<br>0.955 (0.018)	5.6 (4.0 - 16.4)<br>6.3 (3.0)
ROBEX (original)	5	0.936 (0.877 - 0.952)<br>0.93 (0.017)	0.992 (0.895 - 0.999)<br>0.983 (0.026)	0.96 (0.927 - 0.969)<br>0.958 (0.011)	5.5 (4.0 - 19.1)<br>6.7 (3.5)
SynthStrip (original)	1	0.953 (0.924 - 0.971)<br>0.952 (0.01)	0.921 (0.862 - 0.972)<br>0.922 (0.021)	0.997 (0.995 - 0.999)<br>0.997 (0.001)	4.0 (2.3 - 8.0)<br>4.4 (0.7)
SynthStrip (original)	2	0.961 (0.928 - 0.974)<br>0.959 (0.01)	0.932 (0.87 - 0.976)<br>0.931 (0.021)	0.997 (0.99 - 0.999)<br>0.997 (0.002)	4.0 (4.0 - 7.4)<br>4.4 (0.7)
SynthStrip (original)	3	0.968 (0.945 - 0.978)<br>0.967 (0.007)	0.953 (0.905 - 0.977)<br>0.951 (0.016)	0.995 (0.989 - 0.997)<br>0.995 (0.002)	4.0 (3.0 - 5.2)<br>4.0 (0.4)

SynthStrip (original)	4	0.973 (0.945 - 0.979)<br>0.968 (0.01)	0.962 (0.903 - 0.98)<br>0.954 (0.023)	0.995 (0.99 - 0.999)<br>0.995 (0.002)	4.0 (2.7 - 5.2)<br>4.0 (0.5)
SynthStrip (original)	5	0.973 (0.961 - 0.981)<br>0.971 (0.005)	0.966 (0.935 - 0.986)<br>0.964 (0.013)	0.994 (0.987 - 0.998)<br>0.993 (0.002)	4.0 (2.5 - 4.1)<br>3.9 (0.3)
SynthStrip-noCSF (original)	1	0.926 (0.89 - 0.951)<br>0.925 (0.014)	0.866 (0.804 - 0.915)<br>0.865 (0.025)	0.999 (0.997 - 1.0)<br>0.999 (0.001)	8.3 (4.6 - 21.3)<br>9.6 (3.6)
SynthStrip-noCSF (original)	2	0.937 (0.901 - 0.954)<br>0.933 (0.015)	0.883 (0.821 - 0.918)<br>0.877 (0.026)	0.999 (0.998 - 1.0)<br>0.999 (0.0)	8.0 (5.1 - 20.0)<br>8.6 (3.0)
SynthStrip-noCSF (original)	3	0.945 (0.912 - 0.959)<br>0.944 (0.009)	0.897 (0.841 - 0.925)<br>0.896 (0.017)	0.999 (0.999 - 1.0)<br>0.999 (0.0)	6.9 (4.4 - 15.0)<br>7.5 (2.3)
SynthStrip-noCSF (original)	4	0.938 (0.911 - 0.958)<br>0.939 (0.012)	0.886 (0.837 - 0.923)<br>0.887 (0.022)	1.0 (0.999 - 1.0)<br>0.999 (0.0)	8.0 (5.3 - 14.1)<br>7.9 (1.9)
SynthStrip-noCSF (original)	5	0.943 (0.921 - 0.954)<br>0.941 (0.008)	0.893 (0.855 - 0.914)<br>0.891 (0.015)	1.0 (0.998 - 1.0)<br>0.999 (0.0)	8.0 (4.5 - 15.9)<br>8.5 (2.6)
d-SynthStrip (original)	1	0.942 (0.908 - 0.971)<br>0.942 (0.014)	0.895 (0.835 - 0.953)<br>0.896 (0.026)	0.999 (0.997 - 1.0)<br>0.999 (0.001)	4.3 (4.0 - 8.0)<br>4.9 (1.1)
d-SynthStrip (original)	2	0.958 (0.924 - 0.971)<br>0.954 (0.012)	0.926 (0.859 - 0.957)<br>0.919 (0.024)	0.998 (0.995 - 1.0)<br>0.998 (0.001)	4.2 (4.0 - 7.1)<br>4.6 (0.9)
d-SynthStrip (original)	3	0.967 (0.938 - 0.974)<br>0.965 (0.009)	0.945 (0.891 - 0.963)<br>0.941 (0.018)	0.997 (0.994 - 0.999)<br>0.997 (0.001)	4.0 (3.6 - 5.8)<br>4.2 (0.4)
d-SynthStrip (original)	4	0.967 (0.952 - 0.975)<br>0.966 (0.007)	0.946 (0.916 - 0.962)<br>0.944 (0.015)	0.996 (0.994 - 0.999)<br>0.997 (0.001)	4.0 (4.0 - 4.6)<br>4.1 (0.2)
d-SynthStrip (original)	5	0.969 (0.951 - 0.978)<br>0.967 (0.006)	0.947 (0.911 - 0.965)<br>0.945 (0.014)	0.997 (0.995 - 0.999)<br>0.997 (0.001)	4.0 (4.0 - 6.3)<br>4.2 (0.5)
