# Supplementary material for: Skull stripping tools in pediatric T2-weighted MRI scans: a retrospective evaluation of segmentation performance
Source: Front Neurosci. 2025 Dec 18;19:1715514. doi: 10.3389/fnins.2025.1715514 (PMC12756446; doi:10.3389/fnins.2025.1715514)
Supplement: Supplementary file 2 [file Data_Sheet_2.pdf]

Checklist for STARE-HI Reporting Guideline

This checklist was created by Adrian Schulz based on the following publication:

> Talmon J, Ammenwerth E, Brender J, de Keizer N, Nykänen P, Rigby M. STARE-HI--Statement on reporting of evaluation studies in Health Informatics. Int J Med Inform. 2009 Jan;78(1):1-9. doi: 10.1016/j.ijmedinf.2008.09.002.

Direct citation from the publication:

> *"The reader should be aware that STARE-HI is intended as a guideline, not a rigid structural standard. We do recognize that the order of items and the detail to which the various items may be described in a report or paper may depend on the audience, the type of paper, the available space, and the type of study."*

1 Title

**Requirements**	**Check**
Signal Word (evaluation/assessment/study)	☑ Evaluation
Type of System evaluated	☑ Skull Stripping Tools
Study Question	☑ Segmentation Performance
Study Design	☑ Retrospective

2 Abstract

**Content**	**Check**
Objective	☑ Evaluation of skull stripping tools in T2w pediatric MRI scans
Setting (e.g. primary care)	☑ Hannover Medical School
Participants	☑ 199 scans of children under five years with normal brain development and no brain pathologies
Outcome Measures	☑ Dice score, 95th percentile Hausdorff distance, sensitivity, specificity, integrity segmentations of brain structures
Study Design	☑ Retrospective Data analysis
Major Results	☑
Conclusion	☑ SynthStrip is recommended

3 Keywords

Content	Check
Signal Word “Evaluation”	☑
Type of System evaluated	☑ skull stripping
Setting (e.g. primary care)	☒ Not applicable in retrospective analysis
Outcome Measures	☑ Dice score
Study Design	☑ Retrospective Studies

4 Introduction

Content	Check
Scientific Background<br>- Characterization of the system evaluated	 ☑ functionality of brain stripping tools
Rationale for the Study<br>- Reason<br><br>- Context of Evaluation<br>- Stakeholders<br>- Possible influence of the results	 ☑ No evaluation of the tools on pediatric, T2w MRI scans available<br>☑ Brain age prediction<br>☒<br>☒
Objectives of study<br>- Study Questions<br><br>- Hypotheses<br><br>- Ethics Approval	 ☑ finding best-suited skull stripping model<br>☒ implicit in evaluating and comparing different tools<br>☑

5 Study context

Content	Check
Organizational setting<br>- Name<br>- Geographical location<br>- Type (e.g. university hospital)<br>- Department	 ☑ Hannover Medical School<br>☑ Hannover<br>☒ implicit in Hannover Medical School<br>☑ Pediatric Neuroradiology
System details and system in use<br>- Technical description<br>- Context of system use (e.g. laboratory, administrative)<br>- Type of System (e.g. open source, commercial)<br>- Type of information processed<br>- Task supported by system (e.g. documentation)<br>- Duration, purpose and extent of system usage at facility<br>- Number and profession of users of the system at facility<br>- Additional information (e.g. customization)	 ☑ Part of Introduction<br>☑ Whenever skull stripping is needed (see Introduction)<br>☑ Open-source<br><br>☑ T2w MRI-Scans<br>☑ Skull Stripping<br><br>☒ Not applicable to open-source research tools<br>☒ Not applicable to open-source research tools<br>☒ Not explicitly stated that no customization took place

6 Methods

Content	Check
Study Design<br>- Type of Study<br><br>- Reasons for choice<br>- Potential Bias<br><br><br>- How potential Bias is handled	 ☑ retrospective analysis of monocentric clinical routine data<br>☒<br>☑ See Discussion:<br>Monocentric design, Skewed Age Distribution, only healthy individuals<br>☒
Theoretical background<br>- Theories building the base for study choice	 ☒ Not applicable (retrospective analysis of clinical routine data)
Participants<br>- Inclusion Criteria<br><br><br><br>- Exclusion Criteria<br>- Allocation control/intervention<br>- Sample Size<br>- Power Calculation	 ☑ Normal brain Development,<br>Age under five years<br>T2-weighted MRI-scan<br>Informed Consent<br>☑ Relevant motion artifacts<br>☒ Not applicable (study not controlled)<br>☑ n = 199<br>☒ Not applicable (all routine data available meeting inclusion criteria)
Study flow<br>- Beginning and End<br>- Study periods<br>- Time and description of intervention<br><br>- Flow diagram of study	 ☑ 2011 - 2022<br>☒ Not applicable (clinical routine data)<br>☒ Not applicable (study not interventional)<br>☒ Not applicable (study not interventional, no follow up)
Outcome measures or evaluation criteria<br>- outcome measures<br><br><br><br>- Define important concepts	 ☑ Dice score, 95th percentile Hausdorff distance, sensitivity, specificity, integrity segmentations of brain structures<br>☑ Rules for manual ground truth segmentation
Methods for data acquisition and measurement<br>- Data collection<br><br><br>- Methods applied and their outcome measures<br><br><br><br><br>- Validation of methods applied<br>- Retrospective/prospective<br>- Blinding	 <br>☑ Available T2w MRI scans from clinical routine at Hannover Medical School meeting inclusion criteria<br>☑ Dice score, 95th percentile Hausdorff distance, sensitivity, specificity, manual analysis of scans for assessing the integrity of segmentations of brain structures<br>☒<br>☑ retrospective<br>☒

Methods for data analysis<br>- Statistical techniques<br><br>- Analysis methods<br><br><br>- Software product used<br>- Use of triangulation	 ☑ Shapiro-Wilk test, Friedman test, two-sided Wilcoxon signed-rank test<br>☑ Dice score, 95th percentile Hausdorff distance, sensitivity, specificity, manual analysis of scans<br>☑ 3D Slicer, Python, dcm2niix, MeVisLab<br>☒ Not applicable

7 Results

**Content**	**Check**
Demographic and other study coverage data<br>- N<br>- Demographic Data<br>- Clinical Characteristics<br>- Drop-Outs	 ☑ 199<br>☑ Sex, Age distribution<br>☑ Normal Brain Development<br>☒ Not applicable (no Follow Ups)
Unexpected events during the study that influenced design of the study	☒ Not applicable (clinical routine data)
Study findings and outcome data<br>- Absolute numbers<br>- Tables and Figures referenced in text<br>- Emphasis on most important and most striking results in text	 ☑<br>☑<br>☑
Unexpected observations during the study that did not influence design of the study but allowed further insights	☒ Not applicable

8 Discussion

**Content**	**Check**
Answers to study questions	☑ SynthStrip is most suitable model
Strengths and weaknesses of the study<br>- Strength<br>- weaknesses	 ☑ size of cohort, quality of ground truth<br>☑ monocentric, age distribution skewed, only healthy subjects, region splitting based on dimension of ground truth masks
Results in relation to other studies<br>- Novelty of results<br>- Comparability of study designs<br>- Reasons for disagreement	 ☑<br>☑<br>☑
Meaning and generalizability of the study<br>- Meaning for stakeholders<br>- Generalizability/applicability	 ☒<br>☑ Since study is the external validation of different tools, it aims for measuring the generalizability

Unanswered and new questions<br>- New questions<br>- Type of research needed	 ☑ how to address partial volume effect<br>☑ multicentered with pathologies

9 Conclusion

Content	Check
Main Findings	☒
Impact of findings	☒
Recommendation	☑ SynthStrip is recommended
Outlook	☑ Future research and ideas for improving user-friendliness

10 Authors' contribution

Content	Check
AS	☑ Writing – original draft<br>Conceptualization<br>Investigation<br>Software<br>Formal Analysis<br>Visualization
ED	☑ Writing – review & editing,<br>Data curation<br>Formal Analysis
KW	☑ Writing – review & editing<br>Data Curation<br>Software<br>Visualization
AnS	☑ Writing – original draft<br>Software
EB	☑ Writing – review & editing<br>Data Curation<br>Conceptualization<br>Supervision<br>Investigation
DW	☑ Writing – original draft<br>Conceptualization<br>Investigation<br>Supervision<br>Project administration

11 Competing interests

Content	Check
AS	☑ None
ED	☑ None
KW	☑ None
AnS	☑ None
EB	☑ None
DW	☑ None

12 Acknowledgement ☒

13 References ☑

14 Appendices / Supplements ☑
